# The Effect of Overcoming the Digital Divide on Middle Frontal Gyrus Atrophy in Aging Adults: Large-Scale Retrospective Magnetic Resonance Imaging Cohort Study

**DOI:** 10.2196/73360

**Published:** 2025-07-22

**Authors:** Yumeng Li, Xinyue Zhang, Jiaqing Sun, Junying Zhang, Aiqin Zhu, Xin Li, Zhanjun Zhang

**Affiliations:** 1 State Key Laboratory of Cognitive Neuroscience and Learning, Beijing Normal University, No.19, Xinjiekouwai Street, Haidian District, Beijing, 100875, China, 86 15810127521; 2 Beijing Aging Brain Rejuvenation Initiative Centre, Beijing Normal University, Beijing, China; 3 Department of Management, London School of Economics and Political Science, London, United Kingdom; 4 Institute of Basic Research in Clinical Medicine, China Academy of Chinese Medical Sciences, Beijing, China; 5 Department of Geriatrics, Qinghai Provincial People’s Hospital, Xining, China

**Keywords:** digital divide, internet use, cognitive aging, neuroplasticity, sMRI, neural decline, structural magnetic resonance imaging

## Abstract

**Background:**

The rapid integration of information technology into daily life has exacerbated the digital divide (DD), particularly among older adults, who often face barriers to technology adoption. Although prior research has linked technology use to cognitive benefits, the long-term neurostructural and cognitive consequences of the DD remain poorly understood.

**Objective:**

The aim of this study is to use large-scale neuroimaging data to examine how the DD affects long-term brain structure and cognitive aging in older adults. It specifically investigates (1) structural and cognitive differences between older adults with and without DD engagement, (2) predictive relationships between group-distinctive brain regions and cognitive outcomes, and (3) longitudinal impacts of DD exposure on accelerated aging trajectories of neural substrates and cognitive functions.

**Methods:**

The study included 1280 community-dwelling older adults (aged 65‐90 y) who completed comprehensive cognitive assessments and structural magnetic resonance imaging scans at baseline. Longitudinal data were available for 689 participants (mean follow-up 3.2 y). Participants were classified into the DD (n=640) and overcoming DD (n=640) groups using rigorous propensity score matching to control for age, education, gender, and baseline health conditions. A computational framework using the searchlight technique and cross-validation classification model investigated group differences in structural features and cognitive representation. The aging rate of each voxel’s structural feature was calculated to explore the long-term influence of the DD.

**Results:**

The DD group showed significant deficits in executive function (*t*=4.75; *P*<.001; Cohen *d*=0.38) and processing speed (*t*=4.62; *P*<.001; Cohen *d*=0.37) compared to the overcoming DD group. Reduced gray matter volume in the DD group spanned the fusiform gyrus, hippocampus, parahippocampal gyrus, and superior temporal sulcus (false discovery rate–corrected *P*<.05). The computational framework identified the key structural substrates related to executive function and processing speed, excluding the ventro-orbitofrontal lobe (classification accuracy <0.6). Longitudinal findings highlighted the long-term impact of the DD. The DD group exhibited faster gray matter volume decline in the middle frontal gyrus (*t*=3.95 for the peak voxel in this cluster, false discovery rate–corrected *P*<.05), which mediated 17% of episodic memory decline (*P*=.02).

**Conclusions:**

Older adults who overcome the DD demonstrate preserved gray matter structure and slower cognitive decline, particularly in frontotemporal regions critical for executive function. Our findings underscore that mobile digital interventions should be explored as potential cognitive decline prevention strategies.

## Introduction

Although mobile devices have become some of the most indispensable technologies in modern society, there is still a significant portion of the population that has never used them. This gap between individuals who are adept at using digital information and communication technologies (ICTs) and those who are not is referred to as the digital divide (DD) [1,2]. However, the impact of the absence of these new avenues for connection, information, and communication on our brains and cognitive capacities, as well as that of screen exposure, remains unclear. The effect of the DD is especially notable within the older population, which demonstrates considerably lower levels of internet acceptance and utilization [3,4]. Given the rapid growth of the aging population in China, it is conceivable that the DD will marginalize older individuals from the swiftly progressing society, potentially impeding their future advancement.

The theory of neural plasticity suggests that the structure of the human brain can undergo long-term changes in response to environmental stimuli and situational triggers [5]. The ubiquitous adoption of the internet and information technology has substantially decreased the costs associated with acquiring new knowledge and participating in social interactions [6]. Consequently, it is contended that the neural modifications induced by the internet are beneficial, especially for older adults who are experiencing decline. Studies endorsing the theory of neural plasticity propose that smartphone-based games could potentially alleviate age-related cognitive decline. However, research on smartphone use inducing neural plasticity effects has been limited to approximately 6 months, thus inadequately exploring the enduring impacts of ICT use on older individuals [7-9].

The theory of frontal lobe control and the extended theory of internet addiction suggest that degenerated prefrontal cortical regions could lead to the dysfunction of cognitive control and inhibition, which is the potential physiological basis for problematic online behaviors [10]. Thus, older individuals with notable prefrontal atrophy in the course of neural aging not only fail to derive benefits from ICT use in acquiring cognitive enhancement, but also face a potential decline in neural flexibility. This decline is also recognized as a clinical precursor to problematic network behaviors in the aging brain [11-13]. The duration of engagement with problem networks was found to be significantly associated with both gray matter atrophy and impairment of white matter integrity [14]. Although the study centered on younger age cohorts, its observed neuropathological characteristics could potentially signify a prospective risk of Alzheimer disease. This suggests the emergence of a long-term digital survival paradigm or a novel environmental instigator for Alzheimer disease, potentially by expediting the neurodegenerative process [15].

In sum, the long-term implications of addressing the DD among the older adult population, whether positive or negative, are yet to be substantiated.

To ascertain the long-term implications of addressing the DD among the older population, this study, using a large-sample neuroimaging cohort, will investigate the following: (1) the differences in brain structure and cognitive performance between individuals who overcome the DD and those who do not, (2) whether the identified brain regions that classify the 2 groups will also predict their cognitive performance, and (3) the longitudinal alterations of the aging rate in cognitive function and brain structure caused by the DD.

A relationship may exist between the longitudinal changes in cognitive function and brain structure observed in aging populations and the existence of a DD.

## Methods

### Study Design

The study population was derived from the Beijing Aging Brain Rejuvenation Initiative, a longitudinal neuroimaging initiative investigating aging-related cognitive trajectories. Participants were not prospectively recruited for this specific analysis but constituted a subset of individuals meeting predefined inclusion criteria. Our samples were community-based. They were all recruited voluntarily. The majority of them live in different communities in Beijing. Established at Beijing Normal University in 2008, the Beijing Aging Brain Rejuvenation Initiative has conducted cohort studies based on the registry of a large community population in the greater metropolitan area of Beijing. All of the participants were 50 years or above at the time of baseline enrollment, capable of living independently, without nervous system diseases or psychiatric disorders, with no metal implants or any other contraindications for undergoing magnetic resonance imaging (MRI) within the body, with 6 or more years of formal education. A total of 3380 participants with MRI data were recruited; participants with a Mini-Mental State Examination score below 24 and without the data to quantify the DD were excluded. As a result, only 1400 participants were recruited in this study. Of 1400 participants, 1280 were included in this study based on propensity score matching (PSM). All of the participants who were registered were revisited every 2-3 years. Throughout the follow-up period, factors like bodily metal implants, severe ailments, loss to follow-up, or participant refusal led to only 689 individuals undergoing a subsequent MRI scan (see Figure S1 in Multimedia Appendix 1 for precise dropout rates and detailed characterization of the longitudinal sample and data collection framework).

### Participants

According to the cross-sectional data, we divided the participants into the DD group and overcoming DD (ODD) group based on the quantification of the DD (see the Measurements section). Power calculations were conducted a priori to ensure adequate statistical sensitivity. For the primary comparison between the digital engagement groups (DD vs ODD), we estimated required sample sizes using a 2-sample *t* test framework with medium effect size (Cohen *d*=.3), 80% power, and *α*=.05: *n*_secondary_=2 × ((*Z*_1−*α*/2_+*Z*_1−*β*_)/d)^2^=2 × (1.96+0.84)^2^=352 per group.

This calculation guided our longitudinal tracking cohort design (n=350 per group) and cross-sectional neuroimaging subsample (n=700 per group). The effect size threshold (*d*=0.3) was selected based on prior neuroimaging studies of technology-related structural plasticity, while the α level (0.05) and power (0.80) followed field-standard conventions [16]. To verify that the cross-group differences of brain structure between the 2 groups were not due to demographic variables (age, gender, and education) and some chronic diseases (hypertension, diabetes, and hyperlipidemia), we used the PSM method [17] to match the DD group with the ODD group based on age, education level, gender, hypertension, diabetes, and hyperlipemia. Following PSM matching, the initial participant count of 1400 decreased to 1280 (each group consisted of 640 participants). The detailed demographic data for both groups can be found in Table S1 in Multimedia Appendix 1. A total of 689 individuals formed the baseline for tracking data, with 296 in the DD group and 393 in the ODD group, both tracked only once. There were no significant demographic differences between the 2 groups at baseline. Detailed data can be found in Table S2 in Multimedia Appendix 1.

### Measurements

#### Quantifying the DD

The quantification indicator of the DD involves an item about the frequency of using ICTs from the Leisure Activity Scale: “How often do you use a computer and mobile devices?” We classified individuals with scores of 0 (never), 1 (≥once per year), and 2 (≥once per month) into the DD group and individuals with scores of 4 (≥once per week) and 5 (everyday) into the ODD group [18-24]. The DD group means failing to overcome the DD while the ODD group means overcoming the DD. We selected usage frequency as the metric to operationalize the DD based on 2 compelling empirical rationales. First, China’s 2023 National Aging Population Survey revealed that 61% of adults aged ≥65 years report never using smartphones, establishing a clinically significant dichotomy between digital adopters and nonadopters within this vulnerable demographic. Second, our *Journal of Medical Internet Research*–published cohort study [25] demonstrated distinct bimodal utilization patterns: 49% exhibited complete digital exclusion (“Never Users”), while 51% engaged in at least occasional usage (“Occasional Users” or higher). This polarized distribution aligns with national surveillance data showing similar bifurcated technology adoption trends among older adults, thereby validating our methodological approach for capturing population-level digital inequities.

In the longitudinal data analysis, participants for whom the use of ICTs could be tracked over time were categorized into either the DD group or the ODD group. We excluded participants who transitioned their status of using ICTs because crossing the DD is a relatively stable state that would not change in the short term. If a transition occurred, it may be due to uncontrollable external factors, which are not the focus of this study.

#### Cognitive Measurements

As described in our previous study, all participants underwent a battery of neuropsychological tests at baseline recruitment [26]. The assessment involved general cognitive ability and cognitive function across 5 domains including memory, language, attention, spatial processing, and executive function. General cognitive ability was tested using the Chinese version of the Mini-Mental State Examination [27]. Memory was tested using the Auditory Verbal Learning Test [28] and the Rey-Osterrich Complex Figure Test [29]. Executive function was tested using the Stroop Color Word Test and the Trail Making Test Part B [30]. Spatial processing was assessed using the Clock Drawing Test [31] and the RO_Copy test [29]. Attention was evaluated using the Symbol Digit Modification Test [32] and the Trail Making Test Part A [30]. Language was tested using the Boston Naming Test and the Verbal Fluency Test (VFT) [33]. The Chinese version and English version of the questionnaire can be found in Multimedia Appendix 2 and Multimedia Appendix 3, respectively.

#### Synthesize the Aggregate Scores for Different Cognitive Domains

We conducted a confirmatory factor analysis analysis for the performance of each cognitive domain (Figure S2 in Multimedia Appendix 1). The path coefficients were used as weights to output the synthesized scores of each cognitive domain. The reason for this processing is that it facilitates subsequent modeling with neuroimage data. As a result, all the neuropsychological tests were divided into 6 domains including memory, visual-spatial, processing speed, executive function, working memory, and language. It should be noted that, due to the fact that only the VFT was included among language-related tests, the confirmatory factor analysis model in this study did not incorporate it. Therefore, the subsequent scores related to language only used the standardized scores of the VFT.

### MRI Image Acquisition and Data Processing

MRI data were acquired using a SIEMENS PRISMA 3T scanner at the Imaging Center for Brain Research at Beijing Normal University during the baseline recruitment and at follow-up several years later. Participants were in a supine position with their heads snugly fixed by straps and foam pads to minimize head movement. The T1-weighted structural images were acquired using 3D magnetization-prepared rapid gradient echo sequences (192 sagittal slices, repetition time=2530 ms, echo time=2.27 ms, slice thickness=1 mm, flip angle=7°, and field of view=256 mm × 256 mm).

The MATLAB2021b [34] and SPM12 [35] toolboxes with default parameters were used to preprocess the structural images. The modulated gray matter images were smoothed with a Gaussian kernel of 8 mm full width at half maximum. We used the mean gray matter map (threshold=0.2) of all the participants to obtain a group brain mask, as well as for subsequent analysis.

High-resolution T1 structural image data were processed using the Cat12 toolbox [36]. Apart from using the East Asian brain template for registration, all other parameters were set to default. The specific processing steps are as follows. The raw data were converted to the NIfTI (Neuroimaging Informatics Technology Initiative) format and underwent tissue segmentation within an individual space to obtain images of gray matter, white matter, and cerebrospinal fluid. After improving segmentation accuracy through affine processing, the images were spatially normalized using high-dimensional DARTEL and geodesic shooting methods [37], registering them to the standard Montreal Neurological Institute brain template after 6 iterations and resampling the images to a voxel size of 1.5 mm³. The original voxel size of the MRI data was 1 × 1 × 1 mm³ [37]. Further local adaptive segmentation corrected local deviations in individual gray matter tissues. Following the assessment of image quality and tissue segmentation effects, all brain tissue images underwent spatial smoothing using a Gaussian kernel function (full width at half maximum=8mm).

### Statistical Analysis

#### The Effect of the DD on Cognitive Function

The intergroup variations between the DD and ODD groups were assessed through an independent samples *t* test. Cohen *d* was used for the calculation of effect size.

The mixed linear model (MLM) was used to examine the influence of the DD variable on the rate of cognitive aging at an individual level. Initially, we established the null model and unconditional growth model. The null model was used to determine the hierarchical structure of the longitudinal data for different cognitive functions, which was suitable for MLM analysis. The unconditional growth model was used to identify significant aging patterns in various cognitive functions over time. After selecting these 2 models, we constructed the full model that encompassed level 1 (described the individual cognitive level aging patterns) and level 2 (investigated the influence of the DD variable on the aging patterns of multiple cognitive abilities in individuals).

Level 1:


$$Cognitive\;Score=\pi_{0}+\pi_{1}(Time)+e$$

Level 2:


$$\pi_{0}=\beta_{00}+\beta_{01}(Age_{baseline})+\beta_{02}(Gender)+\beta_{03}(Edu)+\beta_{04}(Digital\;Divide)+r_{0}$$

$$\pi_{1}=\beta_{10}+\beta_{11}(Age_{baseline})+\beta_{12}(Gender)+\beta_{13}(Edu)+\beta_{14}(Digital\;Divide)+r_{1}$$

The Statistical Package for Social Science (version 25.0; IBM Corp) was used for descriptive statistics and difference analysis. The Mplus (version 8.3) software was used to build an MLM for each domain of cognition. An α of .05 was applied to indicate statistical significance.

#### The Effect of the DD on Brain Structural Characteristics

##### Cross-Sectional Data

We used a 2-sample *t* test on the DD group and ODD group while regressing age, gender, educational level, and total intracranial volume. We used a significance level of *P*<.05 following false discovery rate (FDR) correction to identify group variances, which were then used for subsequent analyses.

**Figure 1.**
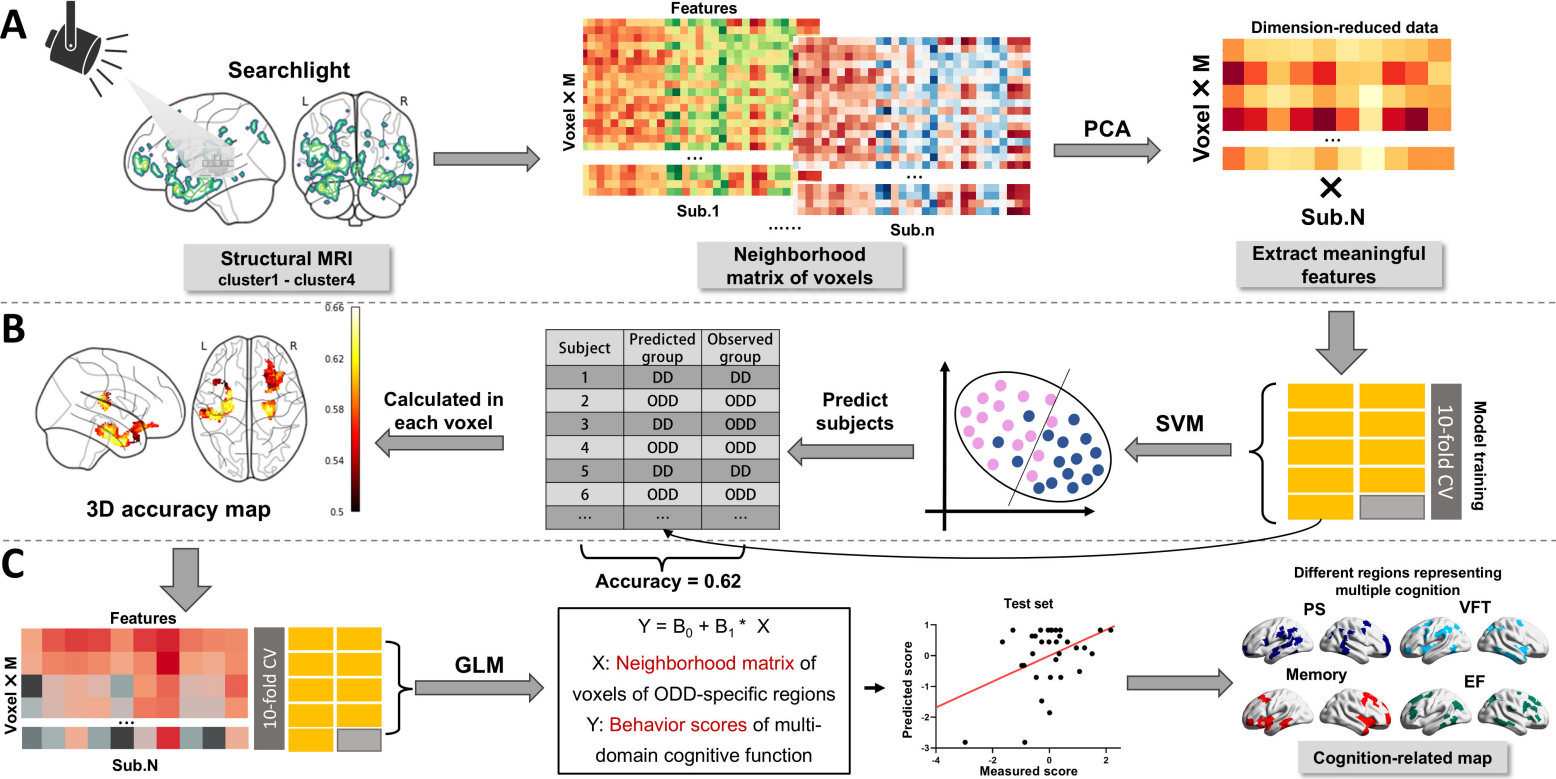
An illustration of the statistical framework using the searchlight technique. (**A**) Searchlight analysis generated a neighborhood voxel matrix using a sliding spherical window. PCA was then applied to reduce data dimensions and extract meaningful features. (B) SVM was used for voxel-based group classification with 10-fold cross-validation. Distinct brain regions with high classification accuracy were identified in a 3D accuracy map using a specified threshold. (**C**) The features were added to a GLM to predict multidomain cognitive function scores. Correlations between predicted and observed scores were calculated to assess domain-specific representation. DD: digital divide; EF: executive function; GLM: general linear model; MRI: magnetic resonance imaging; ODD: overcoming the digital divide; PCA: principal component analysis; PS: processing speed; SVM: support vector machine; VFT: Verbal Fluency Test.

A scheme of multivoxel pattern analysis based on support vector machines (SVM) was used to constrain the brain regions representing the group differences of the DD and predicting the individual’s cognitive performance. The whole statistical framework is illustrated in Figure 1. First, we used searchlight analysis to generate a neighborhood matrix of voxels using a sliding spherical window centered on a specific voxel with the radius of 2 mm. Subsequently, principal component analysis was applied on this matrix to reduce the dimensions of the data to retain 80% of the variance to extract meaningful features (Figure 1A). Second, SVM was used to classify the different groups in each voxel. The classification accuracy determined through 10-fold cross-validation served as a measure of the discriminating capability of the centered voxel. The specific parameter settings of the SVM in MATLAB’s fitcsvm function are as follows by default. We used the linear kernel function as the kernel function and the value of 1 as the regularization parameter (BoxConstraint), which controls the penalty for misclassification. Distinct brain regions exhibiting outstanding classification performance were identified in a 3D accuracy map using a specified threshold (greater than 0.6; Figure 1B). Finally, the dominant regions for classifying different groups also used the searchlight technique to generate a voxel matrix and extract distinct features. These features were then incorporated in a general linear model to predict the behavioral scores of multidomain cognitive function. The correlation between the predicted and observed scores was calculated to delineate the measure of representing the specific domain of cognition. The statistical significance of the correlation was determined using 10,000 permutations, where predictive scores were shuffled for each permutation, and a correlation coefficient was computed. A null distribution was constructed based on the 10,000 correlation coefficients, and multiple comparisons correction was executed with an FDR threshold of *P*<.001. The brain regions with significant correlation coefficients were characterized as regions associated with the DD that represent specific cognitive functions.

##### Longitudinal Data

Based on this, we calculated the annual decline rate of gray matter volume (GMV) for each participant at the voxel level and region level (Anatomical Automatic Labeling template). The maps of decline rate between the DD group and ODD group were analyzed using 2-sample *t* tests with the baseline demographic variables and total intracranial volume controlled. Finally, to establish whether changes in cognitive performance were associated with changes in the decline rate of GMV, Pearson correlation analysis was conducted.

DPABI software was used for the analysis. An independent samples *t* test was used to compare GMV between the DD group and the ODD group. The FDR method for the multiple comparison correction analysis method was applied for the extraction of image data. The statistical significance threshold for the voxel size was set at 0.05, whereas for the cluster size it was established at 0.001. All primary Matlab codes supporting the rest of the findings of this study (mainly in Figure 1) are available online at [38].

### Ethical Considerations

The study was conducted in accordance with the institutional review board at the Imaging Center for Brain Research at Beijing Normal University (ICBIR_A_0041_002_02) and was approved in March 2015, with waived requirements for additional registration given its observational design and use of anonymized archival data. The protocol was approved by the ethics committee of the State Key Laboratory of Cognitive Neuroscience and Learning, Beijing Normal University. We used STROBE (Strengthening the Reporting of Observational Studies in Epidemiology) as our reporting framework. Written informed consent and sociodemographic information were obtained from the participants before initiating the neuropsychological tests. All participants were reimbursed with daily necessities valued at 20 RMB (approximately US $10) and provided with a free screening report covering multiple domains of cognition as a token of appreciation.

## Results

### Individual Differences in Cognitive Function of the Older Population Under the DD

Due to the demographic information being controlled, an independent sample *t* test was used to indicate the cognitive differences between the 2 groups. Table S2 in Multimedia Appendix 1 presents the differences of multidomain cognitive function between the DD group and ODD group. The cognitive domains with the largest effect size were processing speed (*t*=4.62; *P*<.001; Cohen *d*=0.37) and executive function (*t*=4.75; *P*<.001; Cohen *d*=0.38). The results indicated that the performance of these cognitive functions in the ODD group was better than in the DD group.

### The Differences in Structural Substrates Between the DD and ODD Group

**Figure 2.**
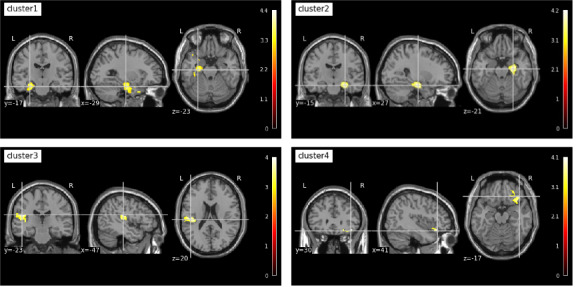
The structural differences between the older adults overcoming the digital divide and those who failed to overcome the divide. Cluster 1 (peak label at temporal pole: *t*=4.41; *P*<.001) and cluster 2 (peak label at hippocampus: *t*=4.19; *P*<.001) included the fusiform gyrus, parahippocampal gyrus, hippocampus, and temporal pole. Cluster 3 (peak label at Rolandic operculum: *t*=4.03; *P*<.001) included the Rolandic operculum, superior temporal gyrus, supramarginal gyrus, and Heschl gyrus. Cluster 4 (peak label at frontal orbital cortex: *t*=4.14; *P*<.001) included the orbital part of the inferior frontal gyrus and part of the insula.

Compared with the DD group, the ODD group showed significantly greater GMV in various brain regions in both hemispheres, mainly located in 4 clusters (Figure 2). The first cluster (peak label at temporal pole: *t*=4.41; *P*<.001) and second cluster (peak label at hippocampus: *t*=4.19; *P*<.001) included the fusiform gyrus, parahippocampal gyrus, hippocampus, and temporal pole. The third cluster (peak label at Rolandic operculum: *t*=4.03; *P*<.001) included the Rolandic operculum, superior temporal gyrus, supramarginal gyrus, and Heschl gyrus. The last cluster (peak label at frontal orbital cortex: *t*=4.14; *P*<.001) included the orbital part of the inferior frontal gyrus and part of the insula. Detailed information for each brain region is listed in Table S3 in Multimedia Appendix 1. These 4 clusters represented the advantageous brain regions for individuals overcoming the digital divide (the ODD group).

### Constrain the Brain Regions Specific to Classifying the ODD Group and the DD Group

**Figure 3.**
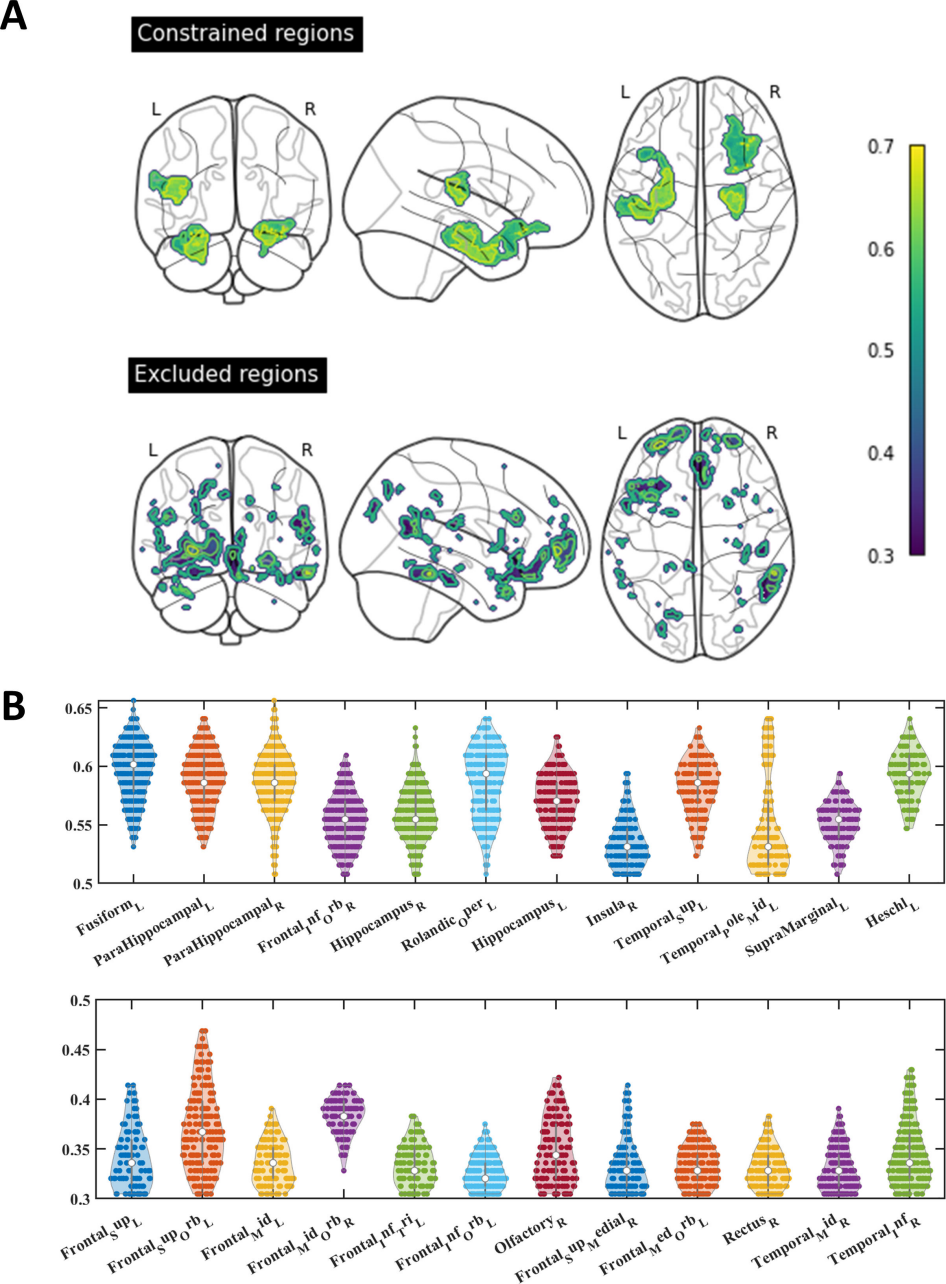
Constraining the brain regions specific for classifying the overcoming the digital divide group and the digital divide group. (**A**) The constrained regions refer to the voxels with classified accuracy above the threshold. The excluded regions are the rest regions that displayed significant gray matter volume difference between the 2 groups; however, the classified accuracy was lower than the threshold. Classified accuracy refers to the accuracy of classifying 2 groups based on voxel-level characteristics through the searchlight statistical framework (see the Methods section). (**B**) The specific distribution and accuracy value of the constrained and excluded regions. The upper map depicts the accuracy distribution of constrained regions, indicating that the average classification accuracy from high to low is the fusiform gyrus, Heschl, Rolandic operculum, parahippocampal gyrus, and hippocampus. The lower map shows the accuracy distribution of the excluded regions, mainly including the inferior frontal regions and orbital frontal regions.

To find out the regions that could effectively represent the features of overcoming the DD, we constructed a statistical framework (Figure 1A) to constrain the regions to classify the structural substrates specific to the DD. The statistical framework analyzed the entire brain to extract structural features from the neighborhood matrix of each voxel. Subsequently, the SVM model was used to predict the state of the DD based on the voxel features. Finally, a 3D accuracy map was generated. The regions where accuracy exceeded a predetermined threshold were called constrained regions and the rest regions were excluded (Figure 3A). The decoding result and the subsequent conjunction analysis both indicated that the excluded regions were mainly involved in the ventromedial orbitofrontal (VMOF) area and parts of the olfactory and temporal lobes were also excluded (Figure 3B).

### Cognitive Representation of the Constrained Regions of DD

**Figure 4.**
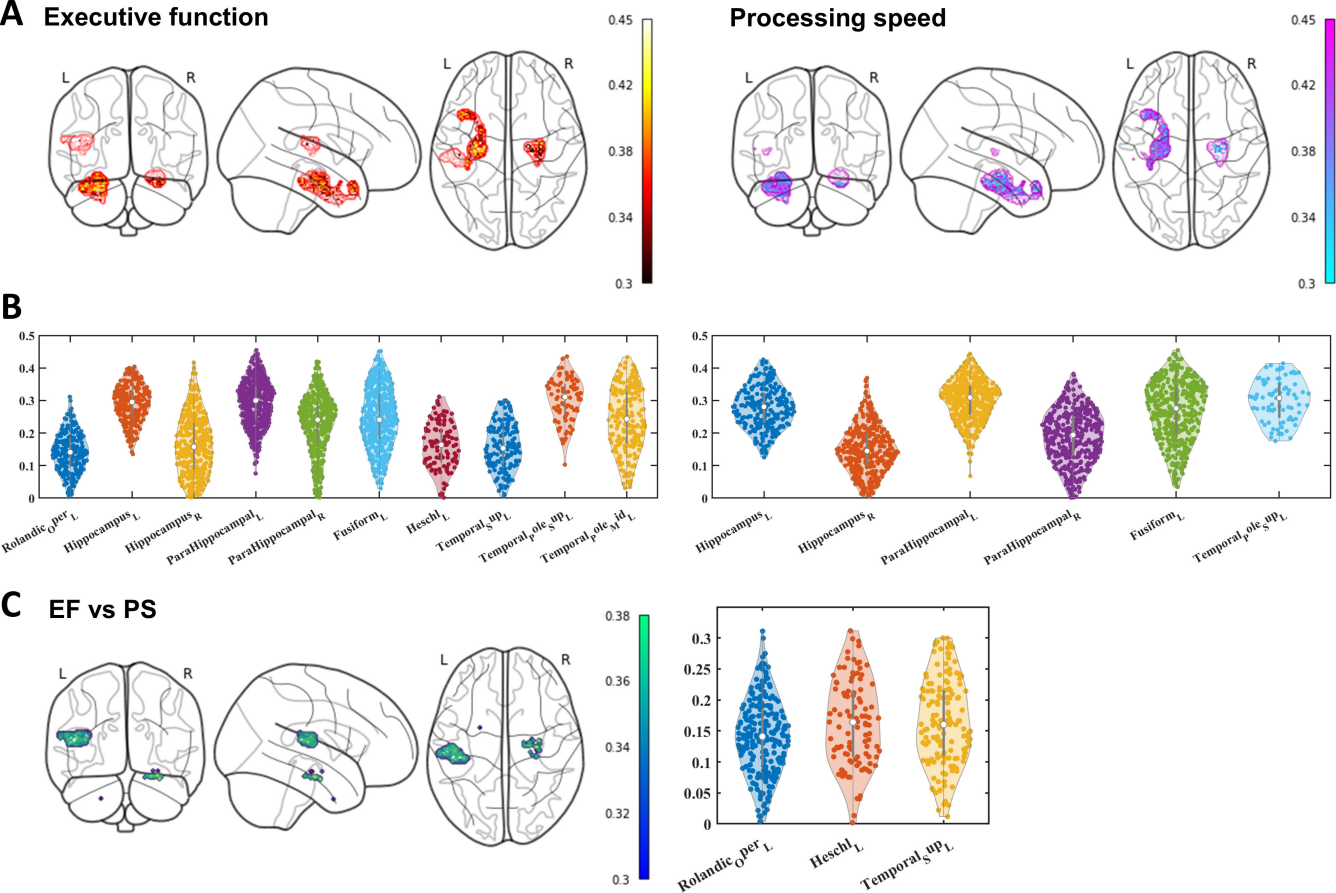
Cognitive representation of the constrained regions of the digital divide. (**A**) The distribution of the constrained regions predicting EF and PS. (**B**) The predictive accuracy(r) of the specific brain region distribution of EF and PS. Combining (A) and (B), we found that the brain areas in the constrained region representing executive function and processing speed are mainly the hippocampus, parahippocampal gyrus, temporal pole, and fusiform gyrus. (**C**) EF regions compared to PS regions: the result indicated the regions specific to EF, including the Rolandic operculum, Heschl gyrus, and superior temporal gyrus, which were decoded as tactile and auditory-related cortices. EF: executive function; PS: processing speed.

Based on the aforementioned framework, we also developed a statistical model to analyze the cognitive representation of these regions (Figure 1C). The findings revealed that the constrained regions are most indicative of executive function, with over 80% of the voxels significantly predicting executive function scores. Processing speed ranked as the second most represented cognitive domain, with approximately 50% of the voxels predicting processing speed scores (Figure 4A). The voxels predicting the rest cognitive functions such as memory, visual-spatial, working memory, and language were no more than 50. This result aligns with behavioral findings that highlight distinct differences between the DD and ODD groups in terms of processing speed and executive function. Notably, the superior temporal pole, hippocampus, and parahippocampal gyrus were identified as critical brain regions involved in these differences (Figure 4B). Moreover, the specific brain regions predicting executive function included the Rolandic operculum, Heschl gyrus, and superior temporal gyrus. These regions were decoded as tactile and auditory-related cortices (Figure 4C).

### Longitudinal Evidence on the DD Influencing Cognitive Aging

Significant interindividual variations were observed for all cognitive abilities in the null model, suggesting the viability of constructing the subsequent MLM (Table S5 in Multimedia Appendix 1). However, in the unconditional growth model, the age-related change trend of visual-spatial and working memory did not reach statistical significance, preventing the construction of the full model (Table S4 in Multimedia Appendix 1). The DD could significantly influence the aging rate of cognitive function (Table S6 in Multimedia Appendix 1). Compared to the ODD group, the memory performance of the DD group displayed a faster aging rate (B_14_=2.54, *P*=.01). Moreover, the participants who were older at baseline also exhibited a more pronounced rate of memory decline (B_11_=−2.47; *P*=.01).

The groups did not differ significantly at baseline in terms of demographic variables, as they were matched. Fewer participants were followed up longitudinally. Thus, we did the independent *t* test for their demographic variables. The result indicated that the longitudinal groups did not differ in terms of these variables (Table S4 in Multimedia Appendix 1).

### The Difference in the Decline Rate of Brain Structure Between the DD and ODD Groups

**Figure 5.**
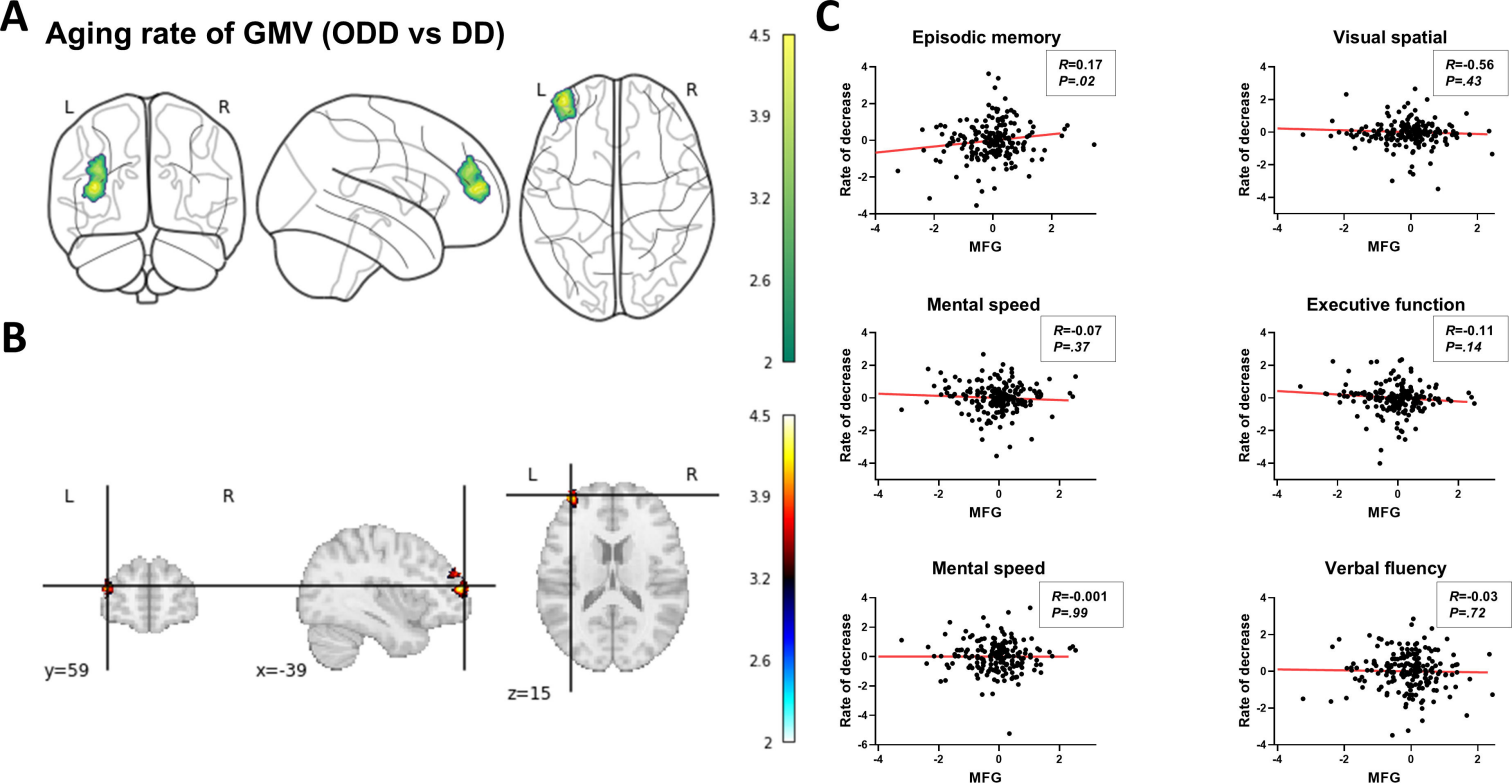
The difference in the decline rate of brain structure between the DD and ODD groups. (A-B) The GMV decline rate in the MFG was significantly lower in the ODD group than in the DD group. (**C**) The decline rate in the MFG significantly correlated with individuals’ memory performance (*R*=0.17; *P*=.02) and showed no significant association with scores in other cognitive domains. DD: digital divide; GMV: gray matter volume; MFG: middle frontal gyrus; ODD: overcoming the digital divide.

To investigate the influence of the DD on structural aging in older adults, the annual aging rate of GMV was calculated for each voxel (see the Statistical Analysis section). We generated brain maps depicting the GMV decline rate for each individual and identified brain regions showing significant group differences in decline rates. The findings revealed that the rate of GMV decline in the middle frontal gyrus (MFG) was notably lower in the ODD group than in the DD group (BA=46; X=−38, Y=53, Z=11; cluster size=719, peak *t* value=3.91; Figure 5A and B). Moreover, this decline rate in the MFG significantly correlated with individual memory performance (*R*=0.17; *P*=.02; Figure 5C) while displaying no significant association with scores in other cognitive domains. Additionally, supplementary materials included a comparison of structural aging rates based on cluster size between the 2 groups. The results indicate significantly lower gray matter aging rates in the MFG, orbitofrontal cortex, and anterior cingulate gyrus in the ODD group as opposed to the DD group, with these rates also correlating with memory scores (Figure S3 and Table S7 in Multimedia Appendix 1).

## Discussion

### Principal Findings

Through the utilization of the searchlight technique in cross-sectional data and integrating it with a cross-validation classification prediction model, this study more precisely constrains the distinct brain regions between the DD and ODD groups. This research not only pinpoints the primary brain regions that exhibit the greatest accuracy in discerning differences between the 2 groups but also delineates how the structural features extracted from these brain regions represent individual cognitive performance. Furthermore, our findings suggest a long-term impact of the DD on brain structure. Specifically, the MFG may exhibit a faster rate of aging related to the episodic memory alternations attributable to the DD.

### Comparison to Prior Work

Through traditional intergroup comparisons, this study observed that the GMV of the ODD group exhibited significant advantages over the DD group in several brain regions, including the fusiform gyrus, hippocampus, parahippocampal gyrus, temporal pole, superior temporal sulcus, and orbitofrontal region. Following the regional screening constraints within the statistical framework of this study, the brain regions capable of more accurately distinguishing between the DD group and ODD group were limited to the fusiform gyrus, hippocampus, parahippocampal gyrus, and a segment of the superior temporal sulcus, while the excluded regions were predominantly concentrated in the VMOF region. The VMOF area is considered a component of the reward circuit [39] and is associated with individual self-control [40].

The Introduction section of this study mentioned a theory suggesting that older adults may experience reduced self-control due to aging of the frontal lobe, which could impede their cognitive benefits from ICTs. However, based on the above results, we do not support this view because the VMOF brain region related to self-control behavior in the frontal lobe shows a low correlation with ICT usage [41], as demonstrated in this study by the inability to properly distinguish the ODD group from the DD group. Moreover, the rationale behind excluding VMOF in our statistical framework is that previous studies have shown, when controlling for other maladaptive behaviors, specific brain regions associated with internet use excluded the VMOF, retaining the regions of the left temporal cortex [42]. Collectively, our findings imply that there is no significant relationship between self-control behaviors of internet use related to reward circuits in the aging population. In contrast to young people, older adults are not easily prone to internet addiction, and older adults who use the internet do not exhibit structural damage of the brain. Moreover, the key brain regions that differentiate between the 2 groups are primarily located around the hippocampus and temporal lobe, aligning with prior research exploring the functional activation patterns associated with internet use in older adults. Older individuals engaging in internet activities exhibit heightened activation in regions like the hippocampus and temporal pole, linked to intricate cognitive processes [43]. These results align with this study, suggesting the potential neural plasticity of these cortical areas associated with advanced cognitive functions in the context of internet utilization. In contrast to prior research, this study did not find a significant contribution of the visual cortex in distinguishing internet usage among older individuals [43-45], but tactile and auditory-related cortices showed notable involvement. This indicates that older individuals rely less on visual stimuli in their internet activities. Additionally, as smartphones advance, managing internet use does not always require visual prompts; for example, individuals can use voice commands and browse web content using the auditory mode. In addition, even simple interactions with the internet via the touchscreen interface of smartphones could lead to sustained neural cognitive changes [46]. This finding aligns with the outcomes of this research, indicating that specific brain regions associated with sensory and tactile functions can differentiate between groups.

The GMV features of each voxel in the aforementioned brain regions are considered effective in distinguishing structural variances between the DD and ODD groups. The rationale for the discriminative capability of these voxels may stem from cognitive-behavioral distinctions linked to their features and the conditions of the DD and ODD groups. Findings from this research indicate that more than 80% of the voxels in the delineating brain regions can predict executive function, with processing speed following closely. These results align with behavioral outcomes, highlighting the predictive capacity of gray matter structural traits in differing cognitive functions between the 2 groups. Notably, brain regions exhibiting relatively strong predictive efficacy encompass the temporal pole, parahippocampal gyrus, and hippocampus. These brain regions are all associated with a range of neurodegenerative diseases and may explain the cognitive impairments found in these diseases.

The temporal pole plays a significant role in the initial pathological progression observed in Alzheimer disease [47]. Notable gray matter deterioration in this region has also been detected during the initial stage of Alzheimer disease [48]. Furthermore, in another study, the degree of temporal pole atrophy was significantly elevated in the mild cognitive impairment group compared to the healthy control group, showing a specific correlation with executive function performance [49]. Similarly, the brain structural features of the parahippocampal gyrus and hippocampus are also associated with the preclinical stages of neurodegenerative diseases [50-52]. Although the decline in these regions is commonly believed to be more related to memory impairments, a significant association was observed between reduced volume in the parahippocampal and hippocampal regions and cognitive decline across various domains, such as episodic memory, working memory, processing speed, and executive function [50,53,54].

The absence of the hippocampus in predicting individuals’ memory performance might appear puzzling at first. However, in this study, this result is reasonable because the computational framework of this study distinguishes the DD and ODD groups based on the structural characteristics of each voxel obtained, and memory is not included in characterizing the cognitive performance differences between the 2 groups. As a result, the prediction of individual cognitive abilities based on brain structure features that differentiate these groups without involving memory aligns with the behavioral findings. Additionally, we adopted the searchlight technique, which is particularly effective for group differentiation [55-57]. Starting from the research question using this method, sacrificing a complete brain region’s structural representation is necessary. The correlation between hippocampal GMV and memory is strongly influenced by hippocampal subfield segmentation [58]. Hence, the limitation in predicting individual memory without accounting for hippocampal segmentation is justifiable.

Initially, we might not instinctively correlate the MFG with memory-related functions. Upon conducting in-depth studies, it has been revealed that the MFG predominantly contributes to attentional functions [59]; moreover, it assumes a more prominent controlling function in the process of semantic integration [60]. Consequently, our initial hypothesis posited that the cognitive functions more closely associated with the decline of the MFG were mental speed, which serves as an indicator of attention, and executive function, reflective of cognitive control. However, the actual correction results indicated that the decline in episodic memory was correlated with the reduction in the GMV of the MFG.

Nevertheless, the corrected data within this study demonstrated that the MFG was linked to the decline in episodic memory, albeit its effect size might confer limited explanatory power (*R*²=2.89%). Notably, extant research on the function of the MFG has predominantly focused on the entire region. Given its spatial proximity to the attention network and the supplementary motor area, the functional interpretations are intertwined [61]. In the current research, the region of the MFG under investigation was closer to the inferior frontal lobe, suggesting that the MFG might be indirectly involved in memory integration via the prefrontal circuitry [62,63]. Moreover, few studies have delved into the relationship between the MFG and its functions from the perspective of age-related decline in the older adult population. Hence, despite the issue of an insufficient effect size, we can still get some enlightenment from it. Age-related declines in episodic retrieval have been associated with volume reductions in the MFG [64-66]. Studies have demonstrated that better structural integrity in the posterior hippocampus and MFG is associated with enhanced within-network connectivity, thus improving associative and source memory performance in older individuals [67]. Therefore, the results of this study show that the slow rate of gray matter decline in the MFG corresponds to lower memory performance decline. However, in longitudinal studies, the increase in MFG activation associated with individuals’ memory decline also corresponded to the longitudinal decrease in the MFG brain structure [68,69]. The structural deterioration of the MFG results in nonbenign functional compensation, linked to reduced cognitive performance and potential brain pathology [70-73]. Hence, the preservation of frontal lobe integrity prompted by older adults engaging with the internet can promote a positive brain function pattern without compromising the frontal lobe’s behavioral control, thereby partially contradicting the hypothesis that age-related frontal lobe inadequacy gives rise to negative online behaviors.

Our study can be further expanded to an alternative perspective, as mobile-device–based cognitive training has emerged as a crucial strategy to address the challenges that dementia poses for cognitive health [74,75]. Nevertheless, due to constraints in clinical randomized controlled trials, the duration of cognitive training interventions typically falls short of 1 year. Moreover, the limited sample size resulting from challenges in recruitment and participant attrition diminishes statistical power, potentially yielding adverse outcomes, particularly concerning gray matter neuroplasticity [76-78]. These factors have sparked debates regarding the efficacy of cognitive training [79,80]. Based on a long-term clinical cohort of big data, this study indicates that the aging population can benefit from the simple use of mobile devices and the internet. Additionally, it revealed consistent gray matter structural variations. Although earlier studies indicated the challenge in reversing gray matter atrophy during aging [81], the rate of gray matter alterations in aging is gradual [82]. Hence, this study proposes a different perspective, suggesting that extended engagement with mobile devices and the internet, leading to increased stimulation and information acquisition for older adults, may offset the progression of gray matter aging to some extent.

### Future Directions

The future development of cognitive decline prevention strategies through mobile devices presents a multifaceted opportunity for advancing personalized health care. For example, neuroplasticity-based cognitive training is an effective way to enhance cognitive function and prevent dementia in healthy older adults. With the spread of the internet and mobile devices, technological advances foster the swift development of computerized cognitive training (CCT), making it possible for older adults to access adaptive multidomain cognitive training in the community or even in their homes. CCT is a convenient and sustainable combination of existing cognitive training and ICTs. Nevertheless, our study revealed that when it comes to leveraging mobile devices to enhance the cognitive abilities of older adults, extensive cognitive engagement on their part might not be necessary. Instead, they merely passively absorb information and, to a certain extent, cooperate through finger movements, which can nonetheless facilitate cognitive improvement. This finding holds significant implications for the future design of more diverse forms of CCT.

However, our study has certain limitations. The conclusion that the causal relationship between overcoming the DD and brain structural preservation remains inadequately established is appropriately considered. Although the longitudinal data provide valuable insights into the correlation between ICT use and slower decline in MFG volume, the study design cannot definitively demonstrate that ICT use is the causal factor. Alternative explanations, such as preexisting cognitive differences that might predispose certain individuals to adopt technology, require more thorough consideration. Therefore, we suggest that future research further explores this correlation.

Based on the above findings and limitations, we recommend the following for future work. First, more rigorous experimental designs can be used to better understand the causal relationship between ICT use and cognitive improvement. Second, it is suggested to conduct further research to identify and control for potential confounding factors such as preexisting cognitive differences. Finally, the exploration of more diverse methods of cognitive enhancement through mobile devices can be expanded to develop more personalized and effective CCT programs.

In summary, while our study provides some evidence of the potential of mobile devices in enhancing the cognitive abilities of older adults, further research is needed to strengthen these conclusions and explore more effective strategies for preventing cognitive decline.

### Limitations

Although this study provides critical insights into the DD’s health impacts, several limitations warrant consideration. First, our operationalization of digital engagement through a single self-reported frequency question (“How often do you use a computer and mobile devices?”) inherently simplifies the multidimensional nature of digital access. Although validated by national surveillance patterns and our prior cohort findings [25], this approach cannot capture critical nuances like device ownership, usage contexts (eg, independent vs proxy-assisted use), or application-specific competencies. Subsequent studies would benefit from multidimensional assessments incorporating device availability (eg, “Do you own internet-enabled devices?”), usage autonomy (“Do you need assistance accessing digital services?”), and functional digital literacy measures. Second, the ubiquitous adoption of the internet and information technology has substantially decreased the costs associated with participating in social interactions; however, this study did not use variables measuring mental health, because only a depression scale was included. In the next stage of our cohort study, we propose to collect more variables of mental health to investigate the social activity deficiencies caused by the DD in the older population. Third, concerning the quantification of the DD, this study did not differentiate between the specific utilizations of ICTs. Some participants may have only passively received phone calls and messages, lacking active engagement with ICTs for accessing further stimuli. Consequently, there remains the potential for misclassification of these individuals into the ODD group. Finally, while PSM was rigorously applied to balance observed covariates between the intervention and control groups, residual confounding from unmeasured variables (eg, socioeconomic status, prior exposure to cognitive training programs, or genetic predisposition) may persist. Future studies should incorporate comprehensive baseline assessments of these potential confounders through multimodal data collection (eg, geocoded socioeconomic indices, detailed lifelong learning histories). Sensitivity analyses using quantitative bias analysis methods could further quantify the potential impact of unmeasured confounding on effect estimates.

### Conclusion

This study provides preliminary evidence that older adults who engage with digital technologies exhibit associations with preserved GMV in Alzheimer disease–vulnerable regions. Our cross-sectional analyses suggest structural differences in the fusiform gyrus, hippocampus, and parahippocampal gyrus between digitally engaged (ODD) and nonengaged (DD) groups, with these regions demonstrating predictive value for executive function and processing speed. Longitudinal observations indicate a correlation between technology use and slower GMV decline in the MFG, though the observational design precludes causal attribution. Although these findings highlight potential neurostructural correlates of digital engagement in aging, alternative explanations—such as preexisting cognitive advantages predisposing individuals to technology adoption—require systematic evaluation. Future intervention studies are needed to clarify whether targeted digital training can modulate GMV trajectories and whether such changes translate to clinically meaningful cognitive preservation.

## Supplementary material

10.2196/73360 Multimedia Appendix 1 Supplementary materials.

10.2196/73360 Multimedia Appendix 2 Elderly Brain Cognitive Ability Assessment Form (original Chinese version).

10.2196/73360 Multimedia Appendix 3 Elderly Brain Cognitive Ability Assessment Form (English version).

## Abbreviations

CCT: computerized cognitive training
DD: digital divide
FDR: false discovery rate
GMV: gray matter volume
ICT: information and communication technologies
MFG: middle frontal gyrus
MLM: mixed linear model
MRI: magnetic resonance imaging
ODD: overcoming digital divide
PSM: propensity score matching
STROBE: Strengthening the Reporting of Observational Studies in Epidemiology
SVM: support vector machines
VFT: Verbal Fluency Test
VMOF: ventromedial orbitofrontal
