# Supplementary material for: The Effect of Overcoming the Digital Divide on Middle Frontal Gyrus Atrophy in Aging Adults: Large-Scale Retrospective Magnetic Resonance Imaging Cohort Study
Source: J Med Internet Res. 2025 Jul 22;27:e73360. doi: 10.2196/73360 (PMC12306509; doi:10.2196/73360)

# Appendix 1 : Supplementary Materials

Supplementary Figure 1. The flowchart illustrating the recruited process of participants
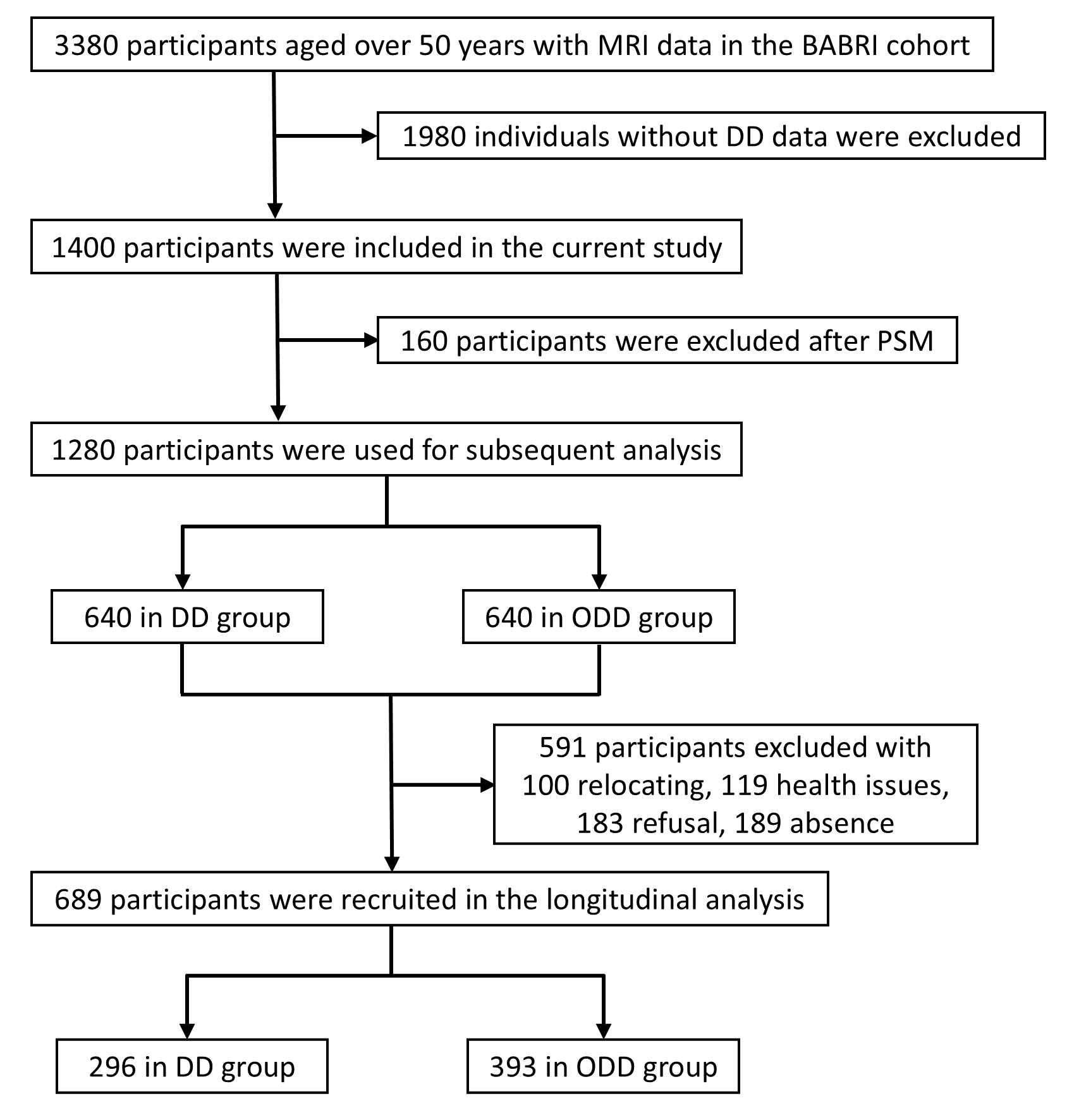


Supplementary Table 1. The demographic information for DD group and ODD group

| Demographic information | DD group | ODD group | t/χ² | *P* |
| --- | --- | --- | --- | --- |
| Gender (M/F) | 244/396 | 234/406 | 0.17 | 0.68 |
| Age (Mean±SD) | 67.30±6.61 | 67.81±7.13 | -0.92 | 0.36 |
| Education | 10.70±2.82 | 10.86±2.83 | -0.73 | 0.47 |
| Hypertension (Yes/No) | 306/334 | 308/332 | 0.06 | 0.97 |
| Diabetes | 168/472 | 168/472 | 0 | 1 |
| Hyperlipidemia | 270/370 | 268/372 | 0.006 | 0.94 |

Supplementary Figure 2. The CFA model of multi-domain of neuropsychological tests


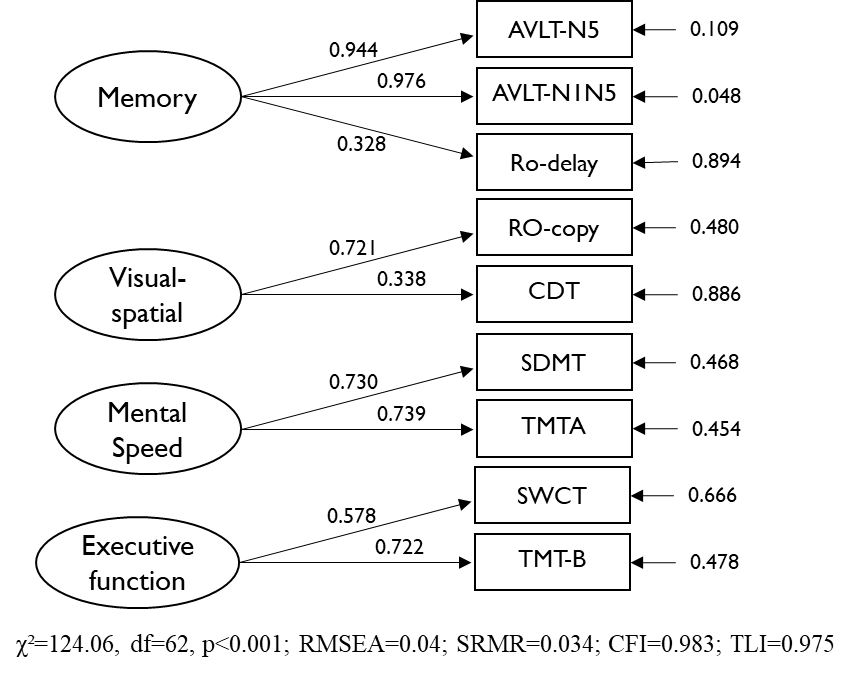


Note: The data below the figure demonstrates the model's goodness of fit, with all metrics indicating a strong fit.

Supplementary Table 2. The cognitive performance for DD group and ODD group

|  | **DD** | **ODD** | **t** | ***P_FDR*** | ***Cohen’s d*** |
| --- | --- | --- | --- | --- | --- |
|  | **n = 640** | **n = 640** |  |  |  |
| Memory | -0.22±2.56 | 2.22±2.96 | 2.00 | 0.046 | 0.16 |
| Visual-spatial | -0.12±0.74 | 0.12±0.97 | 3.55 | <0.001 | 0.28 |
| Processing speed | -0.23±1.32 | 0.23±1.21 | 4.62 | <0.001 | 0.37 |
| Executive function | -0.20±1.24 | 0.20±0.89 | 4.75 | <0.001 | 0.38 |
| Verbal fluency | -0.13±0.99 | 0.13±0.99 | 3.30 | 0.002 | 0.26 |

Note: The p-values in the table have been adjusted by False Discovery Rate (FDR) correction.

Supplementary Table 3. Differences in GMV Between the DD group and ODD group

|  |  | Coordinates | | |  |  |
| --- | --- | --- | --- | --- | --- | --- |
| Brain regions | BA | X | Y | Z | Cluster size | Peak t value |
| DD > ODD |  |  |  |  |  |  |
| cluster 1 |  |  |  |  |  |  |
| Temporal pole | 38 | -39 | 21 | -27 | 222 | 4.41 |
| Fusiform | 20 | -37.5 | -28.5 | -22.5 | 476 | 3.58 |
| Para_hippocampus(L) | 20 | -29 | -17 | -23 | 474 | 3.78 |
| hippocampus(L) | 20 | -29 | -11 | -19 | 262 | 3.38 |
| cluster 2 |  |  |  |  |  |  |
| hippocampus(R) | 20 | 27 | -15 | -21 | 428 | 4.19 |
| Para_hippocampus(R) | 20 | 27 | -12 | -22 | 395 | 3.94 |
| cluster 3 |  |  |  |  |  |  |
| Rolandic_Oper | 48 | -47 | -23 | 20 | 312 | 4.03 |
| SupraMarginal | 48 | -50 | -23 | 19 | 127 | 3.83 |
| Superior Temporal | 48 | -46 | -29 | 12 | 175 | 3.49 |
| cluster 4 |  |  |  |  |  |  |
| Frontal_Inf_Orb | 47 | 41 | 30 | -17 | 415 | 4.14 |
| Insula | 38 | 38 | 18 | -15 | 193 | 3.66 |

Abbreviation: ODD, overcoming the Digital Divide; DD, failed to overcome the Digital Divide; BA, Brodmann area.

Note: The multiple comparison correction was used by the false discovery rate correction. Positive t values indicate the ODD group is higher compared with the DD group.

Supplementary Table 4. The demographic information of longitudinal data for DD group and ODD group

| **Demographic**  **information** | **DD group** | **ODD group** | **t/χ²** | ***P*** |
| --- | --- | --- | --- | --- |
| **Gender (M/F)** | 124/172 | 138/255 | 3.29 | 0.08 |
| **Age (Mean±SD)** | 65.32±5.59 | 66.14±5.68 | -1.89 | 0.06 |
| **Education** | 10.66±2.74 | 10.71±2.85 | -0.24 | 0.81 |
| **Hypertension (Yes/No)** | 148/148 | 195/198 | 0.01 | 0.92 |
| **Diabetes** | 72/224 | 108/285 | 0.87 | 0.35 |
| **Hyperlipidemia** | 140/156 | 204/189 | 1.44 | 0.23 |

The groups did not differ significantly at baseline in terms of demographic variables, as they were matched. Fewer participants were followed up longitudinally. Thus, we did the independent t test for their demographic variables. The result indicated that the longitudinal groups did not differ in terms of these variables.

Supplementary Table 5. Estimation of random effects in the null model and unconditional growth model

|  | **The Null Model** | | **The unconditional growth model** | | |
| --- | --- | --- | --- | --- | --- |
|  | **Within-Variances** | **Between-Variances** | | **Aging rate(π_1_)** |  |
| **Memory** | 8.00^***^ | 7.66^***^ | | -0.027^*^ |  |
| **Processing Speed** | 5.93^***^ | 6.54^***^ | | -0.041^***^ |  |
| **Executive Function** | 5.80^***^ | 5.42^***^ | | -0.032^**^ |  |
| **Visual-spatial** | 3.91^***^ | 3.94^***^ | | -0.002 |  |
| **Verbale fluency** | 4.00^***^ | 6.32^***^ | | -0.022^*^ |  |

The null model was utilized to determine the hierarchical structure of the longitudinal data for different cognitive functions, which was suitable for MLM analysis. The results indicated that the whin-level variability was significant in all domains of cognitive function, suggesting the feasibility of constructing subsequent models.

The unconditional growth model was used to identify significant aging patterns in various cognitive functions over time. The results indicated that most of the cognitive function declined with aging except the visual-spatial(VS) and the working memory(WM). Thus, full models will be established for all cognitive abilities except for VS and WM.

Supplementary Table 6. Longitudinal evidence on the digital divide influencing cognitive aging

| **Aging Rate (B1j)** | | | | | **Initial state (B0j)** | | | |
| --- | --- | --- | --- | --- | --- | --- | --- | --- |
|  | **Age(B_11_)** | **Gender (B_11_)** | **Edu(B_12_)** | **ODD-DD(B_14_)** | **Age(B_01_)** | **Gender (B_02_)** | **Edu(B_03_)** | **ODD-DD(B_04_)** |
| *Episodic memory* | -2.47^*^ | 0.58 | -1.04 | 2.54^*^ | 2.34^*^ | 0.14 | 1.85 | -1.55 |
| *Processing speed* | -2.15^*^ | -0.87 | 0.45 | 0.72 | 0.02 | 0.51 | 0.92 | 0.88 |
| *Executive function* | -3.22^**^ | -0.81 | -0.85 | 1.5 | 0.57 | 0.59 | 1.41 | -0.01 |
| *Language* | -1.43 | -1.76 | -1.18 | 1.65 | -2.02 | 2.75 | 1.22 | -0.11 |

**Notes: the coefficients of MLM(B1j, B0j) refers to the function of Level 1 and Level 2 (See Statistical Analysis)**

Abbreviations: Edu, education level; ODD-DD, the participants remained on overcoming the digital divide during the tracking process compared to the participants remained on failing to overcome the digital divide;

Supplementary Table 7. The difference in the decline rate of cluster-size brain regions between DD and ODD group

| Cognition | t_value | *P_fdr* | Cohen’d |
| --- | --- | --- | --- |
| Frontal_Mid_Orb_R | 2.19 | 0.019 | 0.41 |
| Frontal_Sup_Orb_R | 1.96 | 0.021 | 0.4 |
| Frontal_Inf_Orb_R | 1.95 | 0.023 | 0.38 |
| Cingulum_Ant_L | 1.86 | 0.023 | 0.36 |
| Frontal_Mid_Orb_L | 1.68 | 0.032 | 0.36 |
| Cingulum_Ant_R | 1.55 | 0.032 | 0.31 |
| Frontal_Mid_L | 1.36 | 0.037 | 0.3 |

Supplementary Figure 3. The difference in the decline rate of cluster-size brain regions between DD and ODD group


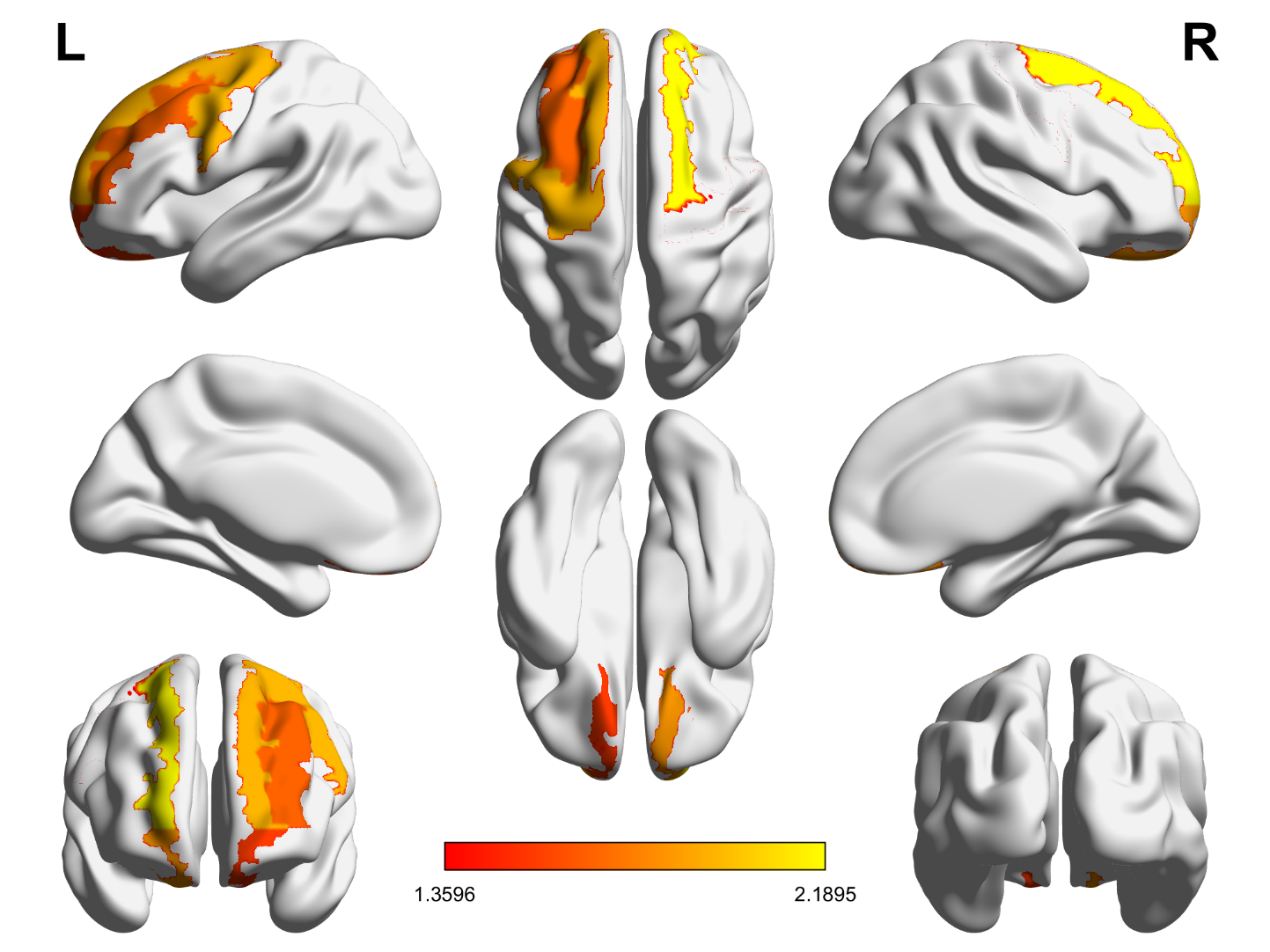

Supplement: Multimedia Appendix 1 [file jmir-v27-e73360-s001.docx]
