# Supplementary material for: The Effect of Overcoming the Digital Divide on Middle Frontal Gyrus Atrophy in Aging Adults: Large-Scale Retrospective Magnetic Resonance Imaging Cohort Study
Source: J Med Internet Res. 2025 Jul 22;27:e73360. doi: 10.2196/73360 (PMC12306509; doi:10.2196/73360)
Supplement: Multimedia Appendix 2 [file jmir-v27-e73360-s002.docx]

# Appendix 2: Elderly Brain Cognitive Ability Assessment Form (Original Chinese Version)

问卷编号：________

**老年脑认知能力评估表**

受访单位名称：____________

受访者姓名：____________

施测者姓名：____________

特殊情况说明：____________

参与后续检查意愿：____________

| 调查 | 基线 | 随访1 | 随访2 | 随访3 | 随访4 | 随访5 | 随访6 |
| --- | --- | --- | --- | --- | --- | --- | --- |
| 日期 |  |  |  |  |  |  |  |

北京师范大学认知神经科学与学习国家重点实验室

北京师范大学老年脑健康研究中心

编制

声明：任何组织或个人不得擅自以任何形式复制或擅自使用本研究的测试工具，否则将依法追究其责任

一、基本情况(demographic variables)

**姓名 性别 年龄 （ 年 月）**

**身份证号码： 左___ 右___ 利手**

**住址 电话**

**记忆主诉：**有无记忆减退主观感受**______** 记忆减退已持续时间**______**

记忆减退出现原因**______** 是否为此感到焦虑**______**

**受访者教育程度：**正规学校教育年份**___________**年（共**______**年）

1.无小学教育；2. 1+私塾或家教；3. 1+成人识字班或夜校；4.小学肆业；

5.小学毕业；6.初中肆业或毕业；7. 高中肆业或毕业；8. 大专以上

**P101身高 cm 体重 kg**

**P102宗教信仰：** 1.佛教 2.伊斯兰教 3.基督教 4.道教 5.其他 6.无

**P103籍贯：** 省（市）

**P104在北京生活时间： 年**

**P105婚姻情况：**1.已婚 2.未婚 3.丧偶 4.离异 5.其他

**P106生活现状：**1.独居 2.与配偶同住 3.与子女同住  4.其他

**P107住房状况：**1.自有住房 2.租房 3.住子女的房 4.其他

**P108目前是：**1.离休人员 2.退休人员 3.在职人员 4.无退休单位人员

**P109离、退休时间： 年 P110退休后是否仍在工作：** 是□ 否□

**P111离、退休前或目前在职的工作单位：**1.行政机关 2.事业单位（含医疗、教育、科研单位等） 3.部队单位 4.石油与化工、电力和通信行业 5.金融业（银行、证券、保险等） 6.其他国有企业 7.私营个体 8.农民 9.其他

**P112离、退休前或目前的职业：**1.科研人员 2.医务人员 3.教师 4.机关、组织、企业等负责人 5.办事人员 6.商业服务人员 7.工人 8.其他

**P113您个人目前的月收入（元）：** 1.500及以下 2.501-1000 3.1001-1500

4.1501-2000 5.2001-2500 6.2501-3000 7.3001-3500 8.3501-4000 9.4001-4500 10.4501-5000 11.5001-5500 12.5501-6000 13.6001及以上

**P114您对自己现在的收支状况如何评价？**

1.宽裕 2.比较宽裕 3.还可以 4.手头较紧 5.手头很紧

**健康状况：**

**现病史及既往史：Present illness and past medical history:**

糖尿病 □ 病程____________ 血糖控制情况________________

规律服药或用胰岛素：否□ 是□

药物名称及服用方法____________________________

高血压 □ 病程____________ 血压控制情况_________

血压最高值_______

规律服药：否□ 是□

药物名称及服用方法____________________________

高血脂 □ 病程____________ 血脂控制情况________________

规律服药：否□ 是□

药物名称及服用方法____________________________

脑血管疾病 □ TIA（短暂性脑缺血发作） □ 脑梗死 □ 脑出血

病程____________ 规律服药：否□ 是□

药物名称及服用方法____________________________

冠心病 □ 病程____________ 规律服药：否□ 是□

药物名称及服用方法____________________________

**P204 是否还患有下列疾病？** 1.其他类型脑血管病（颈动脉斑块或狭窄、脑血管畸形等） 2.头部外伤史 3.老年性精神疾患（抑郁症、焦虑症等） 4.胃肠道疾病  5.呼吸系统疾病 6.肾脏疾病  7.肿瘤 8.疼痛类疾病及其他 9.颈椎病

二、认知能力测查

**1、简易智能状态量表MMSE**（请注意，每道题目都要进行正误判断或记录答案）

| 1.今年的年份? ＿＿年 2.现在是什么季节? ＿＿季节  3.现在是几月? ＿＿月 4.今天是几号? ＿＿日（±1天请记录）  5.今天是星期几? ＿＿ 6.您现在住在哪个城市? ＿＿  7.您现在住在什么区（县）? ＿＿ 8.您现在住在什么街道（乡）?＿＿  9.我们现在是第几层楼? ＿＿ 10.这儿是什么单位? 单位（名称）＿＿ |
| --- |
| 11.现在我说三样东西的名称,在我全部讲完之后,请您重复说一遍,并记住这三样东西, 因为等一下要再问您：“皮球、国旗、树木”。（以第一次答案记分）  皮球＿＿ 国旗＿＿ 树木＿＿ |
| 12.现在请您计算100连续减去7，共计算5次，说出每减一次的得数。  （记录实际回答） 93＿＿86＿＿79＿＿72＿＿65＿＿ |
| 13.（施测者出示手表） 请问这是什么? 手表＿＿  （出示笔）请问这是什么? 笔＿＿ |
| 14.现在我说一句话, 请您按照我说的话清楚地重复一遍，这句话是:  “**四十四只石狮子**”（只说一遍，只有正确、咬字清楚的才记1分）＿＿ |
| 15.请阅读这张卡片所写的句子并照着去做（施测者出示写有“闭上您的眼睛”大字的卡片，如果受访者闭上眼睛，记正确）＿＿ |
| 16.（施测者出示一张纸，说下面一段话后，将纸给被试，不要重复说明，不要示范，顺序需正确）  请用右手拿这张纸＿＿ 把纸对折＿＿ 将纸放在大腿上＿＿ |
| 17.请您说一句完整的、有意义的句子（句子必须有主语、动词）  施测者记下句子＿＿＿＿＿＿＿＿＿＿＿＿＿＿ |
| 18.请您按样子画图。（**画在本页背面**） |
| 19.现在请您告诉我，刚才我要您记住的三样东西是什么?  皮球＿＿ 国旗＿＿ 树木＿＿ |

**2、Rey-O copy 图片模仿**“我现在给您看一幅图画，请您把那幅画画在白纸上”。（**画在第3页背面**，不限时，不要预告要回忆。画得太慢，要告诉被试加快速度；画得太快，要告诉被试仔细地检查一遍。开始4笔用红色，其余线条用黑色笔） **完成时间：_______**

**3、Stroop test**（请注意，将时间和正确个数准确填写在下方空格中）

**卡片A** 指导语：**“从左到右，请您尽量快而正确地读出以下汉字。”**

| 黄 | 红 | 蓝 | 黄 | 绿 | 红 | 蓝 | 红 | 蓝 | 黄 |  |
| --- | --- | --- | --- | --- | --- | --- | --- | --- | --- | --- |
| 蓝 | 黄 | 黄 | 蓝 | 红 | 蓝 | 黄 | 绿 | 绿 | 红 |  |
| 红 | 绿 | 绿 | 红 | 绿 | 绿 | 绿 | 黄 | 红 | 绿 |  |
| 绿 | 蓝 | 蓝 | 黄 | 黄 | 黄 | 红 | 红 | 黄 | 绿 |  |
| 黄 | 红 | 绿 | 黄 | 蓝 | 绿 | 红 | 绿 | 绿 | 蓝 |  |

**卡片B** 指导语：**“从左到右，请您尽量快而正确地读出以下颜色的名称。”**

| 蓝 | 绿 | 红 | 蓝 | 黄 | 绿 | 黄 | 蓝 | 黄 | 红 |  |
| --- | --- | --- | --- | --- | --- | --- | --- | --- | --- | --- |
| 绿 | 蓝 | 绿 | 红 | 绿 | 黄 | 蓝 | 红 | 蓝 | 黄 |  |
| 蓝 | 红 | 蓝 | 绿 | 红 | 黄 | 红 | 蓝 | 绿 | 黄 |  |
| 红 | 黄 | 红 | 蓝 | 绿 | 蓝 | 绿 | 黄 | 蓝 | 黄 |  |
| 红 | 蓝 | 黄 | 红 | 绿 | 蓝 | 黄 | 红 | 蓝 | 黄 |  |

**卡片C** 指导语：**“从左到右，请您尽量快而正确地读出以下颜色的名称，注意不是字的读音。比如第一个读作“绿”，而不是蓝。如果您理解如何回答，请开始好吗？**

| 绿 | 黄 | 蓝 | 绿 | 红 | 黄 | 蓝 | 红 | 蓝 | 绿 |  |
| --- | --- | --- | --- | --- | --- | --- | --- | --- | --- | --- |
| 蓝 | 红 | 绿 | 蓝 | 黄 | 红 | 绿 | 黄 | 红 | 蓝 |  |
| 红 | 蓝 | 黄 | 红 | 蓝 | 绿 | 黄 | 红 | 黄 | 绿 |  |
| 蓝 | 红 | 绿 | 黄 | 红 | 黄 | 蓝 | 绿 | 红 | 绿 |  |
| 黄 | 红 | 蓝 | 绿 | 蓝 | 绿 | 红 | 蓝 | 黄 | 红 |  |

| 指 标 | 卡片1 | 卡片2 | 卡片3 |
| --- | --- | --- | --- |
| 时 间（秒） |  |  |  |
| 正确阅读个数（M=50） |  |  |  |

**4、符号数字转换测验（SDMT）**

指导语：“请您看，此处（指着样本）有1-9几个数字，每个数字都对应着不同的符号。下面的格子里只有数字没有符号，要请您根据数字，将它对应的符号填在下面。我们先来做个练习……”受试者填完练习区后，“现在请您从这里开始（指着）按顺序填写，要尽量快而准确，做完第一排再做下一排。预备，开始！”同时开始计时，受试者填写时若跳格需提醒“不要跳格”，填写错误不提醒，共计90秒钟填写时间后测试结束。

计分：正确填写个数_____ 填写错误个数______ 跳格提醒次数______

**5、Rey-O delay图片, 回忆**“请您将刚才画过的图再次画在白纸上。”

(**画在第5页背面**，不限时，开始4笔用红色笔，其余线条用黑色笔。) **回忆时间：________**

**6、听觉词语学习测验AVLT（N1、N2、N3）**

学习并即刻回忆3次，指导语：

N1：“现在我会给您念一些词语，请您仔细听，我念完之后请您开始回忆，回忆时不需要按顺序。”

N2/N3：“我再给您念一遍这些词语，您再熟悉一下，我念完后您再次回忆。”

（不告诉受试者词语数量，在格子里记录受试者说出的词语顺序，N3、N4结束时都告知后面还要回忆）

| No | 项目 | N1 | N2 | N3 | 间  隔  其它测验  5分钟 | N4 | 间  隔  其它测验  20分钟 | N5 | N6 | | 再 | 认 |
| --- | --- | --- | --- | --- | --- | --- | --- | --- | --- | --- | --- | --- |
| 1 | 大衣 |  |  |  |  |  |  |  | 花  朵  类 | 腊梅 | R士兵 | R长裤 |
| 2 | 司机 |  |  |  |  |  |  |  |  | 海棠 | 纽扣 | R手套 |
| 3 | 海棠 |  |  |  |  |  |  |  |  | 玉兰 | R百合 | 军人 |
| 4 | 木工 |  |  |  |  |  |  |  |  | 百合 | 西装 | R海棠 |
| 5 | 长裤 |  |  |  |  |  |  |  | 职  业  类 | 律师 | 耳环 | 杜鹃 |
| 6 | 百合 |  |  |  |  |  |  |  |  | 司机 | R玉兰 | R 木工 |
| 7 | 头巾 |  |  |  |  |  |  |  |  | 士兵 | 主任 | 牡丹 |
| 8 | 腊梅 |  |  |  |  |  |  |  |  | 木工 | 荷花 | R大衣 |
| 9 | 士兵 |  |  |  |  |  |  |  | 服  饰  类 | 长裤 | R头巾 | 衬衫 |
| 10 | 玉兰 |  |  |  |  |  |  |  |  | 手套 | R司机 | R律师 |
| 11 | 律师 |  |  |  |  |  |  |  |  | 头巾 | 皮鞋 | 校长 |
| 12 | 手套 |  |  |  |  |  |  |  |  | 大衣 | 玉米 | R腊梅 |
| 正确 |  |  |  |  |  |  |  |  |  |  |  |  |
| 插入错误 |  |  |  |  |  |  |  |  |  |  |  |  |
|  |  |  |  |  |  |  |  |  |  |  |  |  |
|  |  |  |  |  |  |  |  |  |  |  |  |  |

**N3完成时间：_______（5分钟后请测N4) N4完成时间：_______（20分钟后请测N5）**

被试回答“大衣、木工、衬衫、木工”4个词语时，次序记录是：大衣1、木工2、4。插入错误：衬衫。尽可能全部记录。

|  | N1 | N2 | N3 | N4 | N5 |
| --- | --- | --- | --- | --- | --- |
| 总数 |  |  |  |  |  |

**7、连线测验TMT**

| 项 目 | Trails练习题 | Trails1测验题 | Trails2练习题 | Trails2测验题 |
| --- | --- | --- | --- | --- |
| 计 时 （秒） |  |  |  |  |
| 错误连接提醒次数 |  |  |  |  |
| 抬笔提醒次数 |  |  |  |  |

**8、听觉词语学习测验（N4）**

自由回忆12个词语，时间控制在1分钟左右，告知**后面还要回忆**这些词语。

**9、画钟测验CDT**

指导语：“请您画一个钟表的表盘，**把数字和指针都标上**，显示的时间是1点50分。”开始先用红色笔，待受试者画完前5笔后（判定是否锚定4个点即可）换用黑色笔。**画在第7页(前一页)背面，竖向作图**。

| 项　目 | 满分 |  | 项　目 | 满分 |  |
| --- | --- | --- | --- | --- | --- |
| 1.锚定“12，3，6，9”四个点 | 4 |  | 8.中央点位置 | 1 |  |
| 2.写出所有数字 | 4 |  | 9.钟面完整 | 1 |  |
| 3.所有数字在钟面圆圈内 | 3 |  | 10.有时针和分针 | 2 |  |
| 4.顺时针排列 | 1 |  | 11.时针指向正确 | 2 |  |
| 5.1-12数字次序 | 1 |  | 12.分针指向正确 | 2 |  |
| 6.“12，3，6，9”分布对称 | 2 |  | 13.分针比时针长 | 2 |  |
| 7.其他8个数字的位置 | 3 |  | 14.时针和分针都有箭头 | 2 |  |

**10、词语流畅性VFT**

语义流畅性：

**动物** 指导语：“请您在一分钟内，尽可能多地说出您所知道的动物名称”

| **1_~_15秒** | **16_~_30秒** |
| --- | --- |
| **31_~_45秒** | **46_~_60秒** |

**水果** 指导语：“请您在一分钟内，尽可能多地说出您所知道的水果名称”

| **1_~_15秒** | **16_~_30秒** |
| --- | --- |
| **31_~_45秒** | **46_~_60秒** |

**蔬菜** 指导语：“请您在一分钟内，尽可能多地说出您所知道的蔬菜名称”

| **1_~_15秒** | **16_~_30秒** |
| --- | --- |
| **31_~_45秒** | **46_~_60秒** |

**11、Boston命名测验BNT**

指导语：“我现在给您看一些图片，请您告诉我这些图片是什么”。不做提示回答和辨认，记录实际回答。

| 图片 | 回答 | 图片 | 回答 | 图片 | 回答 |
| --- | --- | --- | --- | --- | --- |
| 1.树 |  | 11.羽毛球拍 |  | 21.听诊器 |  |
| 2.笔 |  | 12.蜗牛 |  | 22.金字塔 |  |
| 3.剪刀 |  | 13.海马 |  | 23.漏斗 |  |
| 4.花 |  | 14.飞标 |  | 24.手风琴 |  |
| 5.锯子 |  | 15.口琴 |  | 25.圆规 |  |
| 6.扫把 |  | 16.犀牛 |  | 26.三脚架 |  |
| 7.蘑菇 |  | 17.冰屋 |  | 27.钳 |  |
| 8.衣架 |  | 18.仙人掌 |  | 28.花棚 |  |
| 9.轮椅 |  | 19.扶手电梯 |  | 29.量角器 |  |
| 10.骆驼 |  | 20.竖琴 |  | 30.算盘 |  |

**12、听觉词语学习测验（N5、N6、再认）**

如果此时相距N4已经达到20分钟，则做N5，N6，未到就做高山和人脸量表。

再认——击中个数（学过的词语说学过、没有学过的词语说没有学过）：_______

再认——虚报个数（学过的词语说没有学过、没有学过的词语说学过）：_______

**13、生活方式**

**A.闲暇活动：请根据过去的一年内通常情况作答**

|  | 参与频率 | 每天 | 每周  ≥1次 | 每月  ≥1次 | 每年  ≥1次 | 从不 |
| --- | --- | --- | --- | --- | --- | --- |
| P301 | 阅读（报纸、杂志、书籍） | 4 | 3 | 2 | 1 | 0 |
| P302 | 写作 | 4 | 3 | 2 | 1 | 0 |
| P303 | 课程学习（参加各类讲座，上老年大学等） | 4 | 3 | 2 | 1 | 0 |
| P304 | 棋牌活动（下棋、扑克、打麻将） | 4 | 3 | 2 | 1 | 0 |
| P305 | 手工制作（雕刻、编织、刺绣等） | 4 | 3 | 2 | 1 | 0 |
| P306 | 书法、绘画、摄影 | 4 | 3 | 2 | 1 | 0 |
| P307 | 文艺活动（演奏乐器、表演戏曲、唱歌等） | 4 | 3 | 2 | 1 | 0 |
| P308 | 看电视、听广播 | 4 | 3 | 2 | 1 | 0 |
| P309 | 使用电脑和手机（如浏览网页、微信聊天等） | 4 | 3 | 2 | 1 | 0 |
| P310 | 益智类活动（填字游戏、单人纸牌、魔方等） | 4 | 3 | 2 | 1 | 0 |
| P311 | 有氧耐力运动（步行、慢跑、骑车和游泳等） | 4 | 3 | 2 | 1 | 0 |
| P312 | 肌肉耐力和运动（哑铃、沙袋和拉力器等） | 4 | 3 | 2 | 1 | 0 |
| P313 | 灵活性运动（广播操、韵律操、舞蹈等） | 4 | 3 | 2 | 1 | 0 |
| P314 | 中国传统的武术及保健操（太极拳、剑等） | 4 | 3 | 2 | 1 | 0 |
| P315 | 户外活动（爬山、滑雪、采摘、钓鱼等） | 4 | 3 | 2 | 1 | 0 |
| P316 | 旅游 | 4 | 3 | 2 | 1 | 0 |
| P317 | 小组形式体育活动  （足球、保龄球、毽球、网球、乒乓球、高尔夫球等） | 4 | 3 | 2 | 1 | 0 |
| P318 | 种植类活动（花、草、蔬菜，园艺等） | 4 | 3 | 2 | 1 | 0 |
| P319 | 饲养宠物（养鱼、鸟、狗等） | 4 | 3 | 2 | 1 | 0 |
| P320 | 拜访朋友或亲戚 | 4 | 3 | 2 | 1 | 0 |
| P321 | 参加聚会（宗教集会，公益团体组织活动等） | 4 | 3 | 2 | 1 | 0 |
| P322 | 做家务 | 4 | 3 | 2 | 1 | 0 |
| P323 | 照看小孩 | 4 | 3 | 2 | 1 | 0 |
