# Supplementary material for: The Effect of Overcoming the Digital Divide on Middle Frontal Gyrus Atrophy in Aging Adults: Large-Scale Retrospective Magnetic Resonance Imaging Cohort Study
Source: J Med Internet Res. 2025 Jul 22;27:e73360. doi: 10.2196/73360 (PMC12306509; doi:10.2196/73360)
Supplement: Multimedia Appendix 3 [file jmir-v27-e73360-s003.docx]

# Appendix 3: Elderly Brain Cognitive Ability Assessment Form (English Version)

Subject ID: ________

**Elderly Brain Cognitive Ability Assessment Form**

Subject Unit：____________

Subject Name：____________

Researcher Name：____________

Special Notice：____________

Willingness for Subsequent Tests：____________

| Survey | Baseline | Wave 1 | Wave 2 | Wave 3 | Wave 4 | Wave 5 | Wave 6 |
| --- | --- | --- | --- | --- | --- | --- | --- |
| Date |  |  |  |  |  |  |  |

State Key Laboratory of Cognitive Neuroscience and Learning, Beijing Normal University

Beijing Normal University Aging Brain Health Research Center

Disclaimer: No organization or individual may reproduce or use the research tools of this study without authorization. Violators will be held legally responsible.

1、Demographic variables

**Name: __________**

**Gender: ______**

**Age: ____ years, ____ months**

**ID Number: __________**

**Handedness: Left □ / Right □**

**Address: __________**

**Phone: __________**

**Memory Complaint:**

**Subjective feeling of memory decline: Yes □ / No □**

**Duration of memory decline: __________**

**Cause of memory decline: __________**

**Anxiety about memory decline: Yes □ / No □**

**Education Level:**

**Formal schooling years: ________ years**

1. No primary education; 2. 1+ years of private tutoring; 3. 1+ years of adult literacy classes/night school; 4. Primary school dropout; 5. Primary school graduate; 6. Junior high school dropout/graduate; 7. Senior high school dropout/graduate; 8.College or above.

**P101 Height: ____ cm
P102 Weight: ____ kg**

**P102 Religion:**

1. Buddhism 2. Islam 3. Christianity 4. Taoism 5. Other 6. None

**P103 Hometown:** ____ Province/City
**P104 Years lived in Beijing:** ____ years

**P105 Marital Status:**

1. Married 2. Single 3. Widowed 4. Divorced 5. Other

**P106 Living Situation:**

1. Living alone 2. Living with spouse 3. Living with children 4. Other

**P107 Housing Status:**

1. Owned house 2. Rented house 3. Living in children’s house 4. Other

**P108 Current Status:**

1. Retired (government) 2. Retired (general) 3. Employed 4. No retirement benefits

**P109 Retirement Year:** ______
**P110 Working Post-Retirement:** Yes □ / No □

**P111 Pre/Post-Retirement Employer Type:**

1. Government agency 2. Public institution (medical, education, research, etc.)
2. Military unit 4. Petrochemical, power, or telecom industry
3. Finance (banking, securities, insurance) 6. Other state-owned enterprises
4. Private/self-employed 8. Farmer 9. Other

**P112 Pre/Post-Retirement Occupation:**

1. Researcher 2. Medical staff 3. Teacher
2. Manager (government/enterprise) 5. Clerk 6. Commercial service staff
3. Laborer 8. Other

**P113 Monthly Income (RMB):**

1. ≤500 2. 501–1000 3. 1001–1500 4. 1501–2000 5. 2001–2500
2. 2501–3000 7. 3001–3500 8. 3501–4000 9. 4001–4500 10. 4501–5000
3. 5001–5500 12. 5501–6000 13. ≥6001

**P114 Financial Status Evaluation:**

1. Comfortable 2. Relatively comfortable 3. Neutral 4. Tight 5. Very tight

**Health status**

**Present illness and past medical history:**

- **Diabetes □**
  Duration: ________
  Blood sugar control: ________
  Regular medication/insulin: No □ / Yes □
  Medication details: ________
- **Hypertension □**
  Duration: ________
  Blood pressure control: ________
  Highest recorded BP: ________
  Regular medication: No □ / Yes □
  Medication details: ________
- **Hyperlipidemia □**
  Duration: ________
  Lipid control: ________
  Regular medication: No □ / Yes □
  Medication details: ________
- **Cerebrovascular Disease □**
  TIA □ / Cerebral infarction □ / Cerebral hemorrhage □
  Duration: ________
  Regular medication: No □ / Yes □
  Medication details: ________
- **Coronary Heart Disease □**
  Duration: ________
  Regular medication: No □ / Yes □
  Medication details: ________

**P204 Other Diagnosed Conditions:**

1. Other cerebrovascular diseases (carotid plaque, stenosis, etc.) 2. Head trauma history. 3. Geriatric mental disorders (depression, anxiety, etc.) 4. Gastrointestinal diseases 5. Respiratory diseases 6. Kidney diseases 7. Tumor 8. Pain-related diseases 9. Cervical spondylosis

2、Cognitive assessments

**1、Mini-Mental State Examination (MMSE)**

| 1. Current year? ____ 2. Current season? ____ 3. Current month? ____ 4. Current date? ____ (±1 day acceptable) 5. Current day of the week? ____ 6. Current city? ____ 7. Current district/county? ____ 8. Current street/township? ____ 9. Current floor number? ____ 10. Current institution name? ____ |
| --- |
| 11. Repeat three words: **Ball, Flag, Tree** Ball: ____ Flag: ____ Tree: ____ |
| 12. Serial subtraction: 100 – 7 × 5 93 ____ → 86 ____ → 79 ____ → 72 ____ → 65 ____ |
| 13.Object identification:   - Watch: ____ - Pen: ____ |
| 1. Repeat: **"Forty-four stone lions"** ____________ |
| 15. Follow instruction: **"Close your eyes"**  Correct □ / Incorrect □ |
| 16. Follow instructions:   - Use right hand to take paper: ____ - Fold the paper: ____ - Place it on your left lap: ____ |
| 1. Construct a meaningful sentence: _____________________________ |
| 18. Copy a figure (Figure on the back of this page). |
| 19. Recall the three words: Ball: ____ Flag: ____ Tree: ____ |

**2、Rey-O Complex Figure Copy**

"Now I'll show you a picture. Please draw it on a blank sheet of paper." Draw it on the back of page 3. There is no time limit. Don't announce that you will recall it. The drawing is too slow. Tell the subjects to speed up. The drawing is too fast. Tell the subjects to check it carefully. The first 4 strokes are in red and the rest in black. Finish time:**_______**

**3、Stroop test**（Please note that the time and the correct number should be accurately filled in the blanks below.）

**Task A** Instruction: "From left to right, please read the following Chinese characters as quickly and correctly as possible."

| Yellow | Red | Blue | Yellow | Green | Red | Blue | Red | Blue | Yellow |
| --- | --- | --- | --- | --- | --- | --- | --- | --- | --- |
| Blue | Yellow | Yellow | Blue | Red | Blue | Yellow | Green | Green | Red |
| Red | Green | Green | Red | Green | Green | Green | Yellow | Red | Green |
| Green | Blue | Blue | Yellow | Yellow | Yellow | Red | Red | Yellow | Green |
| Yellow | Red | Green | Yellow | Blue | Green | Red | Green | Green | Blue |

**Task B** Instruction: "From left to right, please read the following Chinese characters as quickly and correctly as possible."

| Blue | Green | Red | Blue | Yellow | Green | Yellow | Blue | Yellow | Red |
| --- | --- | --- | --- | --- | --- | --- | --- | --- | --- |
| Green | Blue | Green | Red | Green | Yellow | Blue | Red | Blue | Yellow |
| Blue | Red | Blue | Green | Red | Yellow | Red | Blue | Green | Yellow |
| Red | Yellow | Red | Blue | Green | Blue | Green | Yellow | Blue | Yellow |
| Red | Blue | Yellow | Red | Green | Blue | Yellow | Red | Blue | Yellow |

**Task C** Instruction: "From left to right, please read out the colors of the following characters as quickly and correctly as possible. Note that it's not the pronunciation of the characters." For example, the first one is read as "Green" instead of "Blue". If you understand how to answer, could you please start?

| Green | Yellow | Blue | Green | Red | Yellow | Blue | Red | Blue | Green |
| --- | --- | --- | --- | --- | --- | --- | --- | --- | --- |
| Blue | Red | Green | Blue | Yellow | Red | Green | Yellow | Red | Blue |
| Red | Blue | Yellow | Red | Blue | Green | Yellow | Red | Yellow | Green |
| Blue | Red | Green | Yellow | Red | Yellow | Blue | Green | Red | Green |
| Yellow | Red | Blue | Green | Blue | Green | Red | Blue | Yellow | Red |

| Task | Task 1 | Task 2 | Task 3 |
| --- | --- | --- | --- |
| Record (seconds) |  |  |  |
| Correct count（M=50） |  |  |  |

**4、Symbol Digit Modalities Test（SDMT）**

Instruction: "Please look. Here (pointing to the sample), there are several numbers from 1 to 9, and each number corresponds to a different symbol." The box below contains only numbers and no symbols. Please fill in the corresponding symbol of the number below according to it. Let's do an exercise first... After the subjects finished filling in the practice area, "Now please start from here (pointing) and fill in in order. Try to do it as quickly and accurately as possible. Finish the first row before moving on to the next one." "Ready, start!" The timing starts simultaneously. When the subject is filling in, if they skip a cell, they need to be reminded "Do not skip a cell". If they fill in incorrectly, no reminder will be given. After a total of 90 seconds of filling time, the test ends.

**Correct:** ____ **Errors:** ____ **Skipped Reminders:** ____

**5、Rey-O Delayed Recall**

“Please draw the picture you just drew on the white paper again.”

(Draw on the back of page 5. There is no time limit. Use a Red pen for the first 4 strokes and a black pen for the remaining lines.)

**Recall Time：________**

**6、Auditory Verbal Learning Test (AVLT)
Immediate Recall (N1, N2, N3):**

Learn and recall immediately three times. Instructions:

N1：“Now I'll read some words to you. Please listen carefully. After I finish, please start to recall. There's no need to follow the order when recalling.”

N2/N3：“I'll read these words to you again. Please get familiar with them. After I finish reading, please recall them again.”

（Without informing the subjects of the number of words, record the order of the words they say in the grid. At the end of N3 and N4, inform them that they still need to recall later）

| No | Task | N1 | N2 | N3 | 5 minutes later | N4 | 20 minutes later | N5 | N6 | | Recognition | |
| --- | --- | --- | --- | --- | --- | --- | --- | --- | --- | --- | --- | --- |
| 1 | Coat |  |  |  |  |  |  |  | Flower | Wintersweet | R Soldier | R Trouser |
| 2 | Driver |  |  |  |  |  |  |  |  | Begonia | Button | R Glove |
| 3 | Begonia |  |  |  |  |  |  |  |  | Magnolia | R Lilly | Military |
| 4 | Woodworker |  |  |  |  |  |  |  |  | Lilly | Suit | R Begonia |
| 5 | Trouser |  |  |  |  |  |  |  | Occupation | Lawyer | Earrings | Cuckoo |
| 6 | Lilly |  |  |  |  |  |  |  |  | Driver | R Magnolia | R Woodworker |
| 7 | Headscarf |  |  |  |  |  |  |  |  | Soldier | Director | Peony |
| 8 | Wintersweet |  |  |  |  |  |  |  |  | Woodworker | Lotus | R Coat |
| 9 | Soldier |  |  |  |  |  |  |  | Clothing | Trouser | R Headscarf | Shirt |
| 10 | Magnolia |  |  |  |  |  |  |  |  | Glove | R Driver | R Lawyer |
| 11 | Lawyer |  |  |  |  |  |  |  |  | Headscarf | Shoes | Headmaster |
| 12 | Glove |  |  |  |  |  |  |  |  | Coat | Corn | R Wintersweet |
| Correct |  |  |  |  |  |  |  |  | Correct |  |  |  |
| False |  |  |  |  |  |  |  |  | False |  |  |  |

**N3 Finish time：_______（5minites later N4) N4 Finish time：_______（20minutes later N5）**

When the participants answered the four words "coat, carpenter, shirt, carpenter", the order was recorded as: coat 1, carpenter 2, 4. Insertion error: Shirt. Record as much as possible.

|  | N1 | N2 | N3 | N4 | N5 |
| --- | --- | --- | --- | --- | --- |
| Total |  |  |  |  |  |

**7、Trail Making Test (TMT)**

| Task | Trails  Practice | Trails1 | Trails2  practice | Trails2 |
| --- | --- | --- | --- | --- |
| Records (seconds) |  |  |  |  |
| Error reminders |  |  |  |  |
| Pen lifts |  |  |  |  |

**8、Delayed Recall（N4）**

Recall 12 words freely, with a time limit of about one minute, and inform that you still need to recall these words later.

**9、Clock Drawing Test (CDT)**

Instruction: "Please draw the dial of a clock and mark the numbers and hands. The displayed time is 1:50." Start with a Red pen. After the subject finishes the first 5 strokes (determine whether the 4 dots are anchored), switch to a black pen. Draw it on the back of page 7 (the previous page), and draw it vertically.

| Task | Full mark |  | Task | Full mark |  |
| --- | --- | --- | --- | --- | --- |
| 1. Anchoring “12，3，6，9” | 4 |  | 8. Central point position | 1 |  |
| 2. draw all numbers inside circle | 4 |  | 9. The clock face is intact. | 1 |  |
| 3. All the numbers are within the circle on the clock face | 3 |  | 10. There are hour hands and minute hands | 2 |  |
| 4. Arrange clockwise | 1 |  | 11. The hour hand points correctly. | 2 |  |
| 5. The sequence of numbers from 1 to 12 | 1 |  | 12. The minute hand points correctly | 2 |  |
| 6. The distribution of "12,3,6,9" is symmetrical | 2 |  | 13. The minute hand is longer than the hour hand | 2 |  |
| 7. The positions of the other eight numbers | 3 |  | 14. Both the hour hand and the minute hand have arrows | 2 |  |

**10、Verbal Fluency Test (VFT)**

Semantic fluency

**Animal**

Instruction: "Please name as many animals as you know within one minute."

| **1_~_15 seconds** | **16_~_30 seconds** |
| --- | --- |
| **31_~_45 seconds** | **46_~_60 seconds** |

**Fruits**

Instruction: "Please name as many fruits as you know within one minute."

| **1_~_15 seconds** | **16_~_30 seconds** |
| --- | --- |
| **31_~_45 seconds** | **46_~_60 seconds** |

**Vegetables**

Instruction: "Please name as many vegetables as you know within one minute."

| **1_~_15 seconds** | **16_~_30 seconds** |
| --- | --- |
| **31_~_45 seconds** | **46_~_60 seconds** |

**11、Boston Naming Test (BNT)**

Instruction: "I'll show you some pictures now. Please tell me what these pictures are." Do not give prompt responses or make identifications. Record the actual responses.

| Figure | Response | Figure | Response | Figure | Response |
| --- | --- | --- | --- | --- | --- |
| 1.tree |  | 11. badminton racket |  | 21. stethoscope |  |
| 2.pen |  | 12.snail |  | 22.pyramid |  |
| 3.scissor |  | 13.sea horse |  | 23.funnel |  |
| 4. flower |  | 14.dart |  | 24.accordion |  |
| 5.saw |  | 15.harmonica |  | 25.compass |  |
| 6. broom |  | 16.rhinoceros |  | 26.tripod |  |
| 7.mushroom |  | 17.igloo |  | 27.pliers |  |
| 8.clothes hanger |  | 18.cactus |  | 28.flower shed |  |
| 9.wheelchair |  | 19.escalator |  | 29.protractor |  |
| 10.camel |  | 20.harp |  | 30.abacus |  |

**12、Delayed Recall (N4, N5, Recognition):**

If the distance between N4 and N5 has reached 20 minutes at this point, do N5 and N6. If they haven't arrived yet, do the mountain and face scales.

- Hits (correct recognition): ____
- False alarms (incorrect recognition): ____

**13、Lifestyle**

**A.** **Leisure activities: Please answer based on the usual situation in the past year**

|  | **Frequency** | **Everyday** | **Weekly** | **Monthly** | **Annually** | **Never** |
| --- | --- | --- | --- | --- | --- | --- |
| **P301** | **Reading (newspapers, magazines, books)** | **4** | **3** | **2** | **1** | **0** |
| **P302** | **Writing** | **4** | **3** | **2** | **1** | **0** |
| **P303** | **Course study (attending various lectures, attending senior universities, etc.)** | **4** | **3** | **2** | **1** | **0** |
| **P304** | **Chess and card activities (playing chess, poker, playing mahjong)** | **4** | **3** | **2** | **1** | **0** |
| **P305** | **Handmade (carving, weaving, embroidery, etc.)** | **4** | **3** | **2** | **1** | **0** |
| **P306** | **Calligraphy, painting, photography** | **4** | **3** | **2** | **1** | **0** |
| **P307** | **Cultural and artistic activities (playing Musical Instruments, performing operas, singing, etc.)** | **4** | **3** | **2** | **1** | **0** |
| **P308** | **Watch TV and listen to the radio** | **4** | **3** | **2** | **1** | **0** |
| **P309** | **Using computers and mobile phones (such as browsing web pages, chatting on wechat, etc.)** | **4** | **3** | **2** | **1** | **0** |
| **P310** | **Puzzle activities (crossword puzzles, solitaire, Rubik's Cube, etc.)** | **4** | **3** | **2** | **1** | **0** |
| **P311** | **Aerobic endurance exercises (walking, jogging, cycling and swimming, etc.)** | **4** | **3** | **2** | **1** | **0** |
| **P312** | **Muscle endurance and exercise (dumbbells, sandbags and resistance bands, etc.)** | **4** | **3** | **2** | **1** | **0** |
| **P313** | **Flexible sports (radio calisthenics, rhythmic calisthenics, dance, etc.)** | **4** | **3** | **2** | **1** | **0** |
| **P314** | **Traditional Chinese martial arts and health exercises (such as Tai Chi and swordsmanship)** | **4** | **3** | **2** | **1** | **0** |
| **P315** | **Outdoor activities (mountain climbing, skiing, picking, fishing, etc.)** | **4** | **3** | **2** | **1** | **0** |
| **P316** | **Traveling** | **4** | **3** | **2** | **1** | **0** |
| **P317** | **Group form sports activities (Football, bowling, shuttlecock kicking, tennis, table tennis, golf, etc)** | **4** | **3** | **2** | **1** | **0** |
| **P318** | **Planting activities (flowers, grass, vegetables, gardening, etc.)** | **4** | **3** | **2** | **1** | **0** |
| **P319** | **Keeping pets (fish, birds, dogs, etc.)** | **4** | **3** | **2** | **1** | **0** |
| **P320** | **Visit friends or relatives** | **4** | **3** | **2** | **1** | **0** |
| **P321** | **Attend gatherings (religious gatherings, activities organized by public welfare groups, etc.)** | **4** | **3** | **2** | **1** | **0** |
| **P322** | **Housework** | **4** | **3** | **2** | **1** | **0** |
| **P323** | **Babysitting** | **4** | **3** | **2** | **1** | **0** |
